# A role for Dynlt3 in melanosome movement, distribution, acidity and transfer

**DOI:** 10.1038/s42003-021-01917-5

**Published:** 2021-03-26

**Authors:** Zackie Aktary, Alejandro Conde-Perez, Florian Rambow, Mathilde Di Marco, François Amblard, Ilse Hurbain, Graça Raposo, Cédric Delevoye, Sylvie Coscoy, Lionel Larue

**Affiliations:** 1 Normal and Pathological Development of Melanocytes, Institut Curie, CNRS UMR3347, Inserm U1021, Université PSL, Orsay, France; 2 Signalisation Radiobiologie et Cancer, CNRS UMR3347, Inserm U1021, Université Paris-Saclay, Orsay, France; 3 Equipe Labellisée – Ligue Contre le Cancer, Orsay, France; 4 CNRS UMR144, Structure and Membrane Compartments, Institut Curie, Centre National de la Recherche Scientifique, Paris Sciences & Lettres Research University, Paris, France; 5 Laboratoire Physico-Chimie Curie, Institut Curie, PSL Research University - Sorbonne Universités, UPMC—CNRS, Paris, France; 6 Departments of Bioengineering and Physics, Center for Soft and Living Matter, Institute for Basic Science (IBS), Ulsan National Institute of Science and Technology, Ulsan, South Korea; 7 CNRS UMR144, Cell and Tissue Imaging Facility, Institut Curie, Centre National de la Recherche Scientifique, Paris Sciences & Lettres Research University, Paris, France

**Keywords:** Organelles, Skin diseases, Dynein

## Abstract

Skin pigmentation is dependent on cellular processes including melanosome biogenesis, transport, maturation and transfer to keratinocytes. However, how the cells finely control these processes in space and time to ensure proper pigmentation remains unclear. Here, we show that a component of the cytoplasmic dynein complex, Dynlt3, is required for efficient melanosome transport, acidity and transfer. In *Mus musculus* melanocytes with decreased levels of Dynlt3, pigmented melanosomes undergo a more directional motion, leading to their peripheral location in the cell. Stage IV melanosomes are more acidic, but still heavily pigmented, resulting in a less efficient melanosome transfer. Finally, the level of Dynlt3 is dependent on β-catenin activity, revealing a function of the Wnt/β-catenin signalling pathway during melanocyte and skin pigmentation, by coupling the transport, positioning and acidity of melanosomes required for their transfer.

## Abstract (web summary)

Aktary et al. identify novel roles for the dynein light chain Dynlt3 in melanosome transport, maturation, and transfer to keratinocytes. They also find that the Wnt/βcatenin signalling pathway controls Dynlt3 levels and thus also contributes to the regulation of melanocyte transport and skin pigmentation.

## Introduction

Melanocytes are neural crest-derived cells that are primarily found in the epidermis and hair follicles but are also found in the inner ear, eye, heart, and meninges^1^. These cells contain specialized organelles, known as melanosomes, which are responsible for pigmentation. Melanosomes belong to the lysosome-related organelle (LRO) family and exist in four different stages^2^. Only stage III and IV melanosomes are pigmented. Stage I melanosomes, the most primitive melanosomes, are membrane-bound early endosomal structures that initiate the intraluminal Pmel fibrillation. Pmel, a melanosomal protein, forms a fibrillar network in the lumen of the melanosome, upon which melanin is eventually deposited after its synthesis. The Pmel fibrils are fully formed in stage II melanosomes, following which melanin synthesis begins. In stage III, different melanogenesis enzymes (e.g., Tyrp1, Dct) are transported and inserted into the melanosome limiting membrane to produce melanin, which deposits on the pre-existing Pmel fibrils. Melanin accumulates in stage IV melanosomes that will become heavily pigmented. Melanosomes/melanin are then transferred to keratinocytes through an exocytosis/phagocytosis process to fulfill their primary role in protecting the skin from UV irradiation^3^. Prior to melanosome exocytosis and melanin transfer, melanosomes must acquire a secretory signature through maturation steps that are still poorly understood. Maturation is a complex process that includes the melanosome lumen deacidification or neutralization, the acquisition of specific endosomal components by the melanosomes, and the concomitant removal of melanosome-associated membrane proteins (Vamp7 and most likely Tyrp1) through the generation and release of melanosomal membrane tubules^4–6^.

The intracellular trafficking of melanosomes, similar to other LROs, involves the action of different families of motor proteins. Along the microtubules, melanosomes are transported by both kinesin and dynein motors^7^. Most kinesins move their respective cargoes from the nucleus towards the periphery, with kinesin I and II thought to be involved in this centrifugal melanosome movement^8,9^. Conversely, dynein motors move their cargoes in the opposite direction, centripetally towards the cell center^10^. At the cell periphery, melanosomes are transferred to the actin microfilaments, where the complex consisting of Myosin Va, Rab27a, and Melanophilin move the melanosomes centrifugally^7^ to be transferred. While mutations in these three genes lead to pigmentation defects in humans^11–13^ and in mice^14–16^, to date, no mutation in dynein and kinesin genes has been described in pigmentation.

The cytoplasmic dynein complex is a macromolecular structure composed of six different subunits: heavy chains, intermediate chains, light intermediate chains, and three families of light chains, including Dynlt3, all of which are present in two copies^17^. This protein complex is activated by binding to different cofactor proteins, such as dynactin^18^. The heavy chains comprise the globular domain which physically interacts with microtubules to generate forward motion through ATP hydrolysis. The intermediate, light intermediate, and light chains interact either directly or indirectly with the heavy chains, bind to activating cofactors such as dynactin, and have a role in cargo recognition and specificity^19,20^. While all of the specific dynein proteins involved in the recognition of melanosomes remain unclear, previous studies using human melanocytes in culture have demonstrated that the cytoplasmic heavy chain (DYNC1H1) and a 74 kDa intermediate chain are expressed in melanocytes and are implicated in melanosome centripetal movement, whereas studies using *Xenopus* melanophores have implicated the light intermediate chain 1 dynein (Dync1li1) in these processes^21–23^.

Dynlt3 belongs to the Tctex family of light chains and is homologous to Dynlt1, although these two family members are not often expressed in the same tissues^24,25^. While no intracellular cargo has been identified for Dynlt3, it is known to interact with viral proteins during infection (e.g., Herpes Simplex virus capsid protein VP26^26^), Dynlt3 also has a role in the regulation of the spindle checkpoint through its interactions with the checkpoint protein Bub3^27^. Interestingly, Dynlt3 can also be localized to the nucleus, where it interacts with the chromatin remodeler SATB1 and is involved in SATB1-mediated repression of the *BCL2* gene^28^.

β-catenin is a multi-functional protein that is involved in cell–cell adhesion, cell signaling, and transcriptional regulation. At the cell membrane, β-catenin interacts with the cytoplasmic domain of classical cadherins (e.g., E-, N-, P-cadherin) and with α-catenin, and is a pivotal component of the *adherens* junction^29^. The levels of cytoplasmic β-catenin can be affected by a number of signaling pathways, most notably the Wnt pathway^30^. In the nucleus, β-catenin interacts with transcriptional regulators (such as Lef/Tcf, Foxo, Smad^30^) to modulate gene expression. In the melanocyte lineage, it has been shown to directly influence melanocyte differentiation by inducing M-MITF or BRN2 expression^31,32^.

In humans and mice, abnormal skin pigmentation results from a reduction/lack of melanocytes, a defect in melanin synthesis, and/or disrupted melanosome transport/maturation/transfer^33^. Using bcat* mice, which express a mutant, stable β-catenin in the melanocyte lineage, we have previously shown that increased, melanocyte-specific β-catenin activity resulted in a lighter coat color phenotype^34^. Furthermore, loss of β-catenin in the melanocyte lineage, using Tyr::Cre/°; βcat Δex2-6^F/F^ mice, resulted in a major reduction of melanocyte proliferation and a coat color phenotype in mice^35^. While β-catenin clearly affects pigmentation, its role in regulating melanosome dynamics has, until now, remained unknown.

In the current study, using melanocyte cell lines established from the skin of wild-type and bcat* C57BL/6J mice in culture, we observed a marked diminution in the number of pigmented melanosomes in the perinuclear area of bcat* melanocytes. We identified Dynlt3, a member of the cytoplasmic dynein complex, as a downregulated component in bcat* melanocytes compared to wild-type, and as a regulator of melanosome movement, distribution, acidity/maturation, and transfer. Our data demonstrate, for the first time, the fundamental role of Dynlt3 in coat and skin coloration.

## Results

### Melanocytes expressing stable β-catenin display peripheral melanosome distribution

**Fig. 1:**
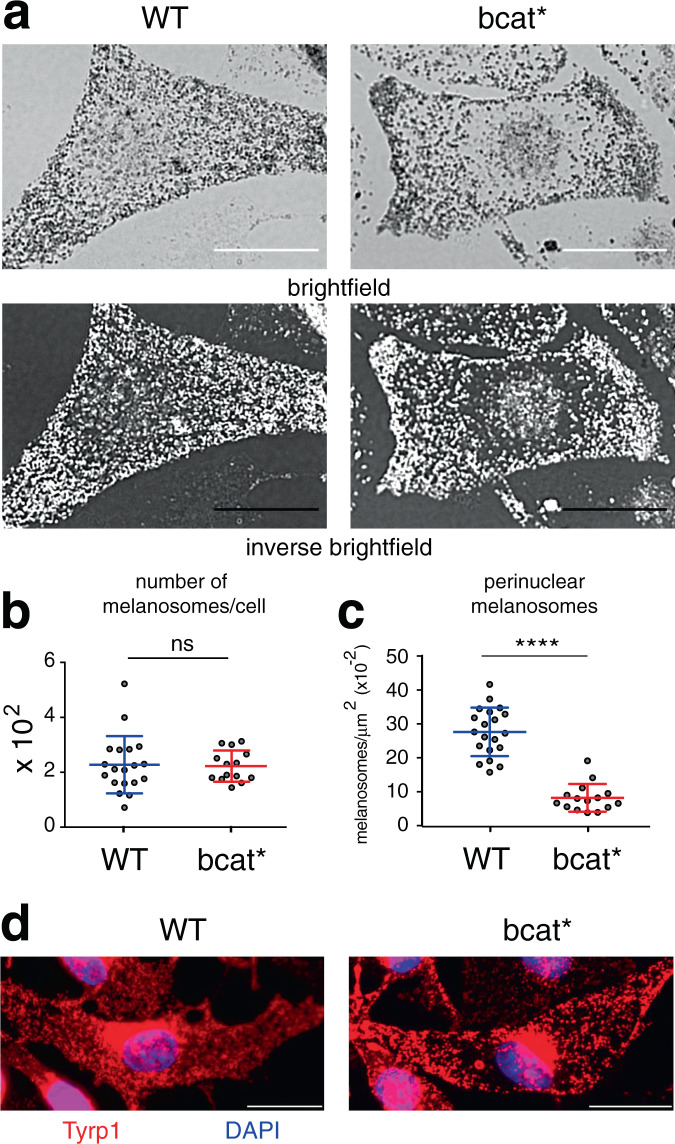
Melanosomes are distributed at the periphery of melanocytes expressing activated β-catenin. **a** Brightfield and inverse brightfield images of WT (9v) and bcat* (10d) melanocytes. In WT cells, 94.1% of cells in culture exhibit a uniform distribution of melanosomes throughout the cell, with only 2.3% of cells having melanosomes absent from the perinuclear area. In contrast, in bcat* cultures, 88.5% of cells show an altered distribution of pigmented melanosomes, with 9.7% of cells having melanosomes evenly distributed throughout the cell. Note that some melanocytes did not have any pigmented melanosomes. Bar, 20 μm. **b** Quantification of the total number of pigmented melanosomes in WT and bcat* melanocytes. Results represent the mean ± the standard deviation (SD) of pooled data from three independent biological experiments with a total of nineteen WT and fourteen bcat* cells. ns signifies no statistical significance (*p* = 0.686) as determined by the two-sided Mann–Whitney test. **c** Quantification of the number of perinuclear pigmented melanosomes in WT and bcat* melanocytes. Results represent the mean ± SD of pooled data from three independent biological experiments with a total of nineteen WT and fourteen bcat* cells. Statistical significance was determined by the two-sided Mann–Whitney test. **** signifies *p* < 0.0001. **d** Immunofluorescence analysis of WT and bcat* melanocytes. Cells were processed for immunofluorescence and stained with anti-Tyrp1 (red) antibodies and the nuclei were stained with DAPI (blue). Note that Tyrp1 staining was also observed in compartments adjacent to the nucleus, which is most likely the protein that is being synthesized and processed in the Golgi/ER. In Supplementary Fig. 1, more examples are shown. Bar, 20 μm.

We established melanocyte cell lines in culture from the skin of wild-type (9v, WT) and bcat* (10d) C57BL/6J pups. As expected, WT pigmented melanosomes (mainly stages III and IV) were distributed uniformly throughout the cell (Fig. 1a, b). However, melanosome localization was markedly different in bcat* melanocytes, which displayed a notable absence of pigmented melanosomes in the perinuclear area (Fig. 1a), while the total number of melanosomes in WT and bcat* cells was similar (Fig. 1b). The quantification of the number of perinuclear melanosomes revealed that the number of pigmented perinuclear melanosomes was approximately three-fold higher in WT cells compared to bcat* cells (Fig. 1c). Immunofluorescence microscopy using anti-Tyrp1 antibodies- mainly labeling stages III and IV melanosomes^36^—further confirmed the reduction of pigmented melanosomes in the perinuclear area of bcat* cells as compared to WT melanocytes (Fig. 1d and Supplementary Fig. 1).

To confirm that the observed reduction of perinuclear melanosomes in this mutant cell line was not limited to the specific bcat* construct, we expressed another active β-catenin mutant, the βcat-Δex3-GFP mutant, in WT melanocytes^37^. This mutant construct encodes for a β-catenin protein lacking the serine and threonine residues that allow for its proteasomal degradation. Similar to the bcat* cells, expression of βcat-Δex3-GFP in WT melanocytes (WT-βcatGFP, fluorescent cells) resulted in a reduced number of perinuclear melanosomes compared to a GFP control (WT-GFP, fluorescent cells) (Supplementary Fig. 2).

Pigmented melanosomes consist of stage III and IV melanosomes. Ultrastructurally, stage III melanosomes contain dark and thick intraluminal melanin-positive fibrils, while stage IV melanosomes are fully filled with pigment. Melanosome maturation was evaluated in WT and bcat* cells by transmission electron microscopy (TEM). Intriguingly, pigmented bcat* melanosomes were significantly larger than WT melanosomes (length: ≈370 vs. ≈300 nm, area: ≈71.8 vs. ≈54.3 nm^2^) (Supplementary Fig. 3).

### β-catenin affects Dynlt3 expression

**Fig. 2:**
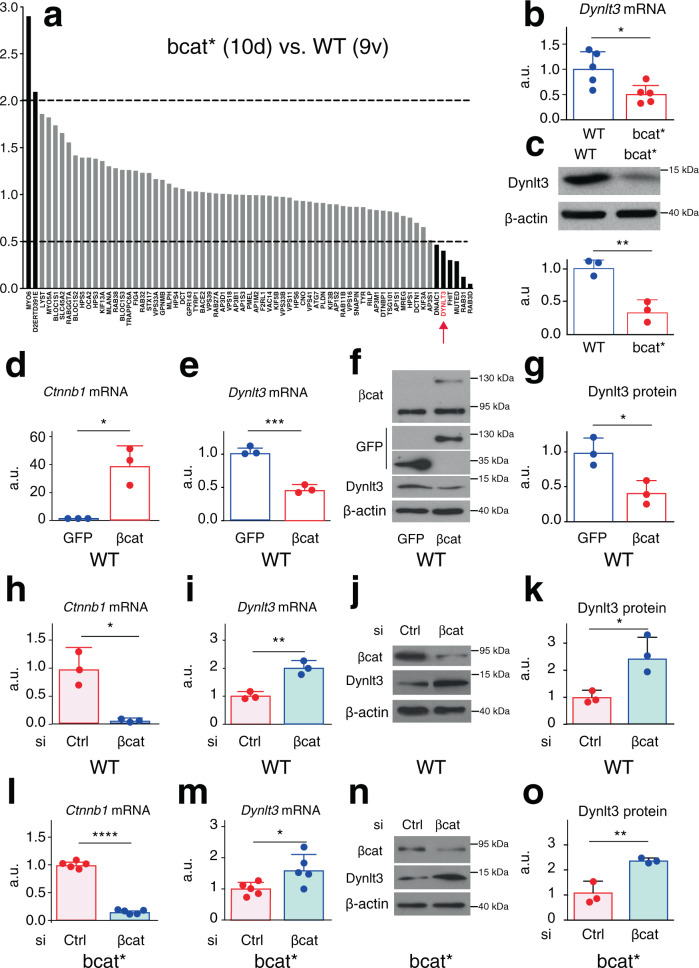
Dynlt3 is downregulated after β-catenin expression. **a** Expression profile of 67 genes associated with the trafficking of melanosomes and other lysosome-related organelles (LRO). Data were obtained from Affymetrix mRNA transcriptomic analyses and is presented as the ratio of expression of bcat* cells to WT cells. **b** RT-qPCR analysis of Dynlt3 mRNA levels in WT and bcat* melanocytes, done from five independent biological experiments. *p = 0.0197. **c** Western blot analysis of Dynlt3 protein levels in WT and bcat* melanocytes. **p = 0.0064. **d**, **e** RT-qPCR analysis of β-catenin (**d**) and Dynlt3 (**e**) expression in WT melanocytes following transfection with the βcat-Δex3-GFP (βcat) expression vector or a GFP control. *p = 0.0104 (**d**) and ***p = 0.0002 (**e**). **f** Western blot of βcat and Dynlt3 levels in WT melanocytes following transfection with the βcat-Δex3-GFP (βcat) expression vector or a GFP control. **g** Quantification of the Dynlt3 western blot data presented in **f**. *p = 0.0213. **h**, **i** RT-qPCR analysis of β-catenin (**h**) and Dynlt3 (**i**) expression in WT melanocytes following transfection with either a control siRNA or siRNA targeting β-catenin. *p = 0.0167 (**h**) and **p = 0.0053 (**i**). **j** Western blot of βcat and Dynlt3 levels in WT melanocytes following transfection with either a control (Ctrl) siRNA or siRNA targeting β-catenin. **k** Quantification of the Dynlt3 western blot data presented in **j**. *p = 0.0387. **l**, **m** RT-qPCR analysis of β-catenin (**l**) and Dynlt3 (**m**) expression in bcat* melanocytes following transfection with either a control siRNA or siRNA targeting β-catenin, done from five independent biological experiments. ****p < 0.0001 (**l**) and *p = 0.0498 (**m**). **n** Western blot of βcat and Dynlt3 levels in bcat* melanocytes following transfection with either a control siRNA or siRNA targeting β-catenin. **o** Quantification of the Dynlt3 western blot data presented in **n**. **p = 0.0088. All statistical significance was determined using unpaired two-sided *t*-tests. For all quantifications, the data is presented as the mean ± SD. Unless otherwise stated, all quantifications of the data were done from three independent biological experiments.

The notable peripheral melanosome distribution in bcat* cells very likely reflected a disruption in their proper intracellular trafficking, due to the exogenous expression of β-catenin. To examine this possibility, we looked at the transcriptome of WT and bcat* melanocytes and specifically focused on the expression of 67 genes known to be involved in the lysosomal-related organelle (LRO) biogenesis, transport, and maturation (Fig. 2a and Supplementary Data 1). From these analyses, we identified Dynlt3, a member of the cytoplasmic dynein complex, as a gene whose expression was decreased two-fold in bcat* cells compared to WT cells.

Transcriptomic data were confirmed by RT-qPCR and western blot analyses and showed that both Dynlt3 mRNA and protein levels were decreased in bcat* cells, relative to WT cells (Fig. 2b, c and Supplementary Fig. 4). These results were corroborated using independent melanocyte cell lines established from independent WT and bcat* pups (Supplementary Fig. 5a). The regulation of Dynlt3 levels by β-catenin was then validated using different approaches. We first overexpressed βcat-Δex3-GFP in WT cells, following which Dynlt3 mRNA and protein levels were both significantly decreased (Fig. 2d–g and Supplementary Fig. 4). Subsequently, we knocked down β-catenin in WT cells and observed an increase in both Dynlt3 mRNA and protein (Fig. 2h–k). Finally, the knockdown of β-catenin in bcat* melanocytes resulted in a similar increase in Dynlt3 mRNA and protein levels (Fig. 2l–o and Supplementary Fig. 4), thereby clearly demonstrating that β-catenin expression appears to have an inhibitory effect on the levels of Dynlt3.

### Knockdown of *Dynlt3* in WT melanocytes phenocopies melanosome distribution in bcat* melanocytes

**Fig. 3:**
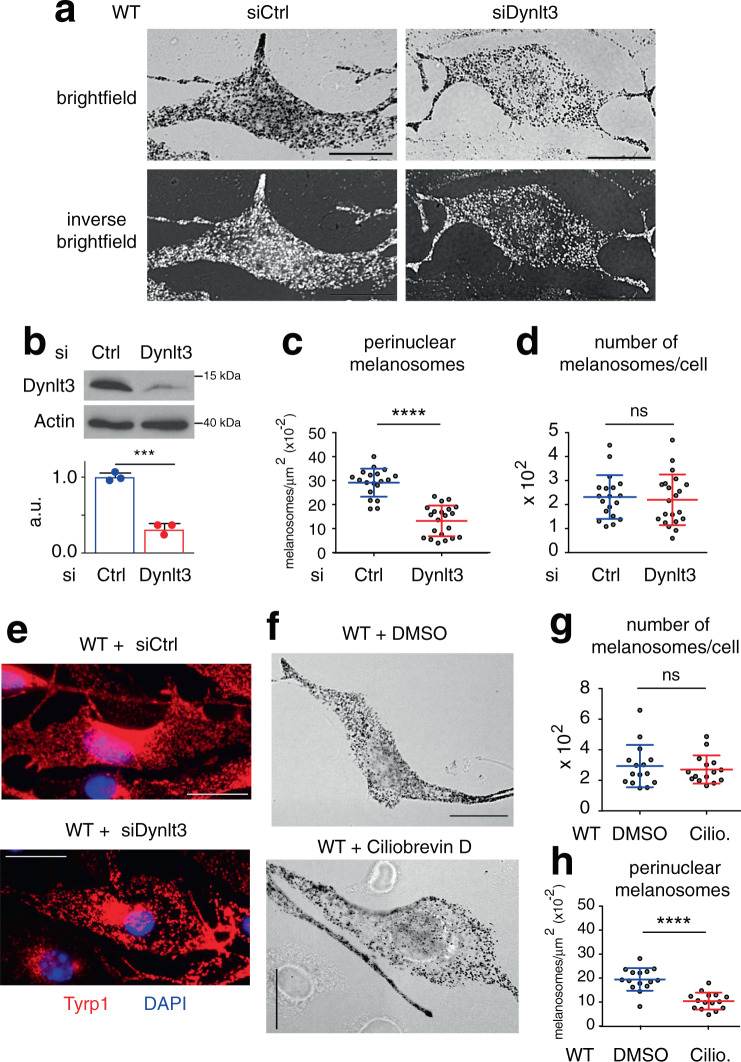
Melanosomes are distributed primarily at the periphery of melanocytes when Dynlt3 levels are decreased. **a** Brightfield and inverse brightfield images of WT cells transfected with either siControl or siDynlt3. Ctrl means control. In WT-siScr cells, 93.6% of cells in culture exhibit a uniform distribution of melanosomes throughout the cell, with only 2.1% of cells having melanosomes absent from the perinuclear area. In contrast, in WT-siDynlt3 cultures, 50.5% of cells show an altered distribution of pigmented melanosomes, with 44.7% of cells having melanosomes evenly distributed throughout the cell. Bar, 20 μm. **b** Western blot analysis of Dynlt3 levels in WT cells transfected with siControl (Ctrl) or siDynlt3. Relative quantification of the western blot analysis was performed from three independent biological experiments, and the mean ± SD is shown. Statistical significance was determined using unpaired two-sided *t*-tests. ***p = 0.0003. **c** Quantification of the number of perinuclear pigmented melanosomes in WT melanocytes transfected with siCtrl or siDynlt3. Results represent the mean ± SD of pooled data from three independent biological experiments with a total of nineteen WT-siCtrl and 21 WT-siDynlt3 cells. Statistical significance was determined by the two-sided Mann–Whitney test, ****p < 0.0001. **d** Quantification of the total number of pigmented melanosomes in WT melanocytes transfected with siCtrl or siDynlt3. Results represent the mean ± SD of pooled data from three independent biological experiments with a total of 19 WT-siCtrl and 21 WT-siDynlt3 cells. Statistical significance was determined by the two-sided Mann–Whitney test, ns: not significant, *p* = 0.7683. **e** Immunofluorescence analysis of WT melanocytes transfected with siCtrl or siDynlt3. Cells were processed for immunofluorescence 48 h after transfection and stained with anti-Tyrp1 (red) antibodies. Nuclei were stained with DAPI (blue). Note that Tyrp1 staining was also observed in compartments adjacent to the nucleus, which is most likely the protein that is being synthesized and processed in the Golgi/ER. In Supplementary Fig. 1, more examples are shown. Scale bar, 20 μm. **f** Brightfield images of WT cells treated with either dimethyl sulfoxide (DMSO) or 80 μM ciliobrevin D for 6 h, after which 9.6% of WT ciliobrevin-treated cells and 1.8% of DMSO-treated cells have decreased perinuclear melanosomes. Bar, 20 μm. **g** Quantification of the total number of pigmented melanosomes in WT melanocytes treated with DMSO or 80 μM ciliobrevin (Cilio) for 6 h. Results represent the mean ± SD of pooled data from three independent biological experiments with a total of 15 cells for each condition. ns signifies no statistical significance (*p* = 0.9759) as determined by the two-sided Mann–Whitney test. **h** Quantification of the number of perinuclear pigmented melanosomes in WT melanocytes treated with DMSO or 80 μM ciliobrevin for 6 h. Results represent the mean ± SD of pooled data from three independent biological experiments with a total of 15 cells for each condition. Statistical significance was determined by the two-sided Mann–Whitney test and **** signifies *p* < 0.0001.

Given that Dynlt3 is a member of the Tctex-type 3 family of Dynein motor light chains implicated in centripetal (retrograde) transport^17,38^ and that its expression was decreased in bcat* cells, we wondered whether Dynlt3 was a player involved in melanosome distribution. To this end, we reduced the levels of *Dynlt3* in WT melanocytes. Similar to bcat* cells, knockdown of *Dynlt3* phenocopied the exclusion of pigmented melanosomes from the perinuclear area (Fig. 3a, b). The number of perinuclear melanosomes was decreased two-fold in WT-siDynlt3 cells as compared to control (Fig. 3c), while the total number of melanosomes was unchanged (Fig. 3d). As previously, WT-siDynlt3 cells stained with an anti-Tyrp1 antibody revealed a notable absence of staining in the perinuclear area, which was not the case in WT-siCtrl melanocytes (Fig. 3e and Supplementary Fig. 1).

We next treated the WT melanocytes with ciliobrevin D, an inhibitor of the cytoplasmic dynein heavy chain, and observed a similar phenotype to bcat* melanocytes and WT melanocytes with decreased levels of Dynlt3. In the ciliobrevin D treated melanocytes, pigmented melanosomes were primarily localized at the cell periphery and were notably less numerous in the perinuclear area (Fig. 3f).

While the ciliobrevin treatment did not affect the total number of melanosomes in WT cells (Fig. 3g), the quantification of the number of perinuclear melanosomes revealed that there was a two-fold decrease in the number of perinuclear pigmented melanosomes when the WT cells were treated with ciliobrevin D (Fig. 3h).

Thus, loss of Dynlt3 in WT melanocytes mimics the effects of disruption of the dynein heavy chain, with respect to pigmented melanosome localization.

**Fig. 4:**
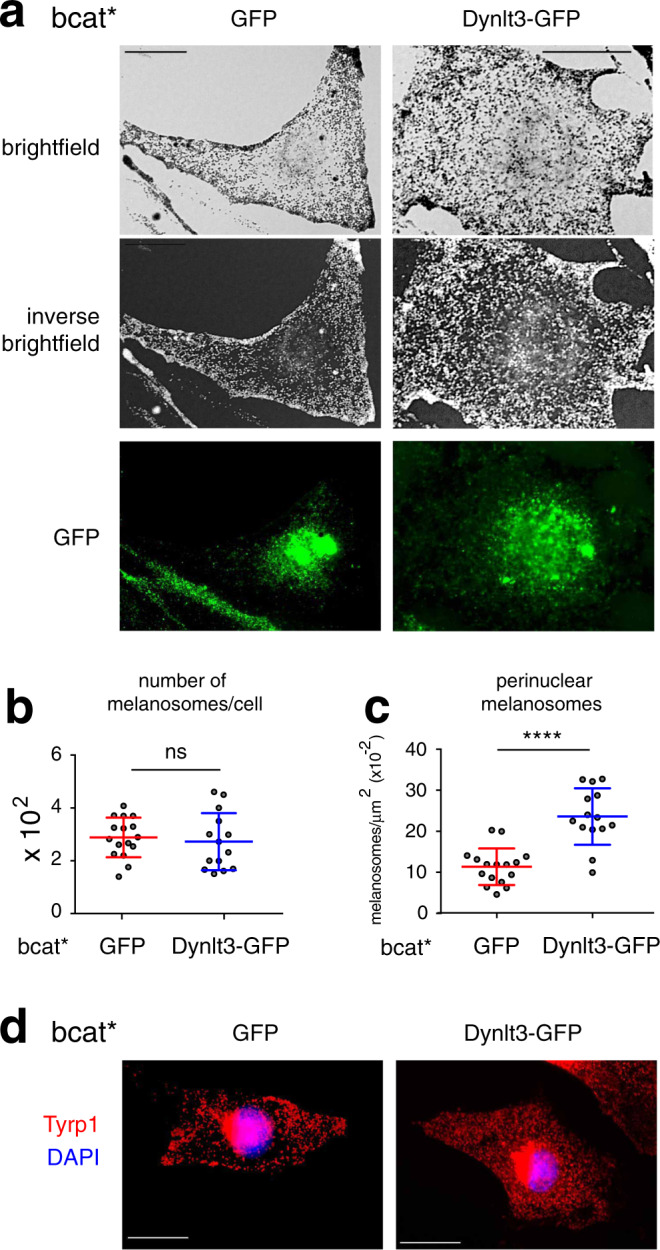
Overexpression of Dynlt3 redistributes melanosomes in bcat* melanocytes. **a** Brightfield, inverse brightfield, and GFP images of bcat* melanocytes transiently expressing GFP or GFP-tagged Dynlt3. Bar, 20 μm. **b** Quantification of the total number of pigmented melanosomes in bcat* melanocytes transfected with expression vector encoding GFP or GFP-tagged-Dynlt3. Results represent the mean ± SD of pooled data from three independent biological experiments with a total of 16 bcat*-GFP and 14 bcat*-Dynlt3-GFP cells. ns signifies no statistical significance (*p* = 0.5178) as determined by the two-sided Mann–Whitney test. **c** Quantification of the number of perinuclear pigmented melanosomes in bcat* melanocytes transfected with expression vector encoding GFP or GFP-tagged-Dynlt3. Results represent the mean ± SD of pooled data from three independent biological experiments with a total of sixteen bcat*-GFP and fourteen bcat*-Dynlt3-GFP cells. Statistical significance was determined by the two-sided Mann–Whitney test and **** signifies *p* < 0.0001. **d** Immunofluorescence analysis of bcat* melanocytes transiently expressing GFP or GFP-tagged Dynlt3. Note the nuclear localization of Dynlt3-GFP, which has been previously observed^28^. Cells were processed for immunofluorescence 48 h after transfection and stained with anti-Tyrp1 (red) antibodies. The nuclei were stained with DAPI (blue). In Supplementary Fig. 1, more examples are shown. Bar, 20 μm.

Finally, to confirm the role of Dynlt3 in rescuing the abnormal melanosome phenotype, we overexpressed Dynlt3 in bcat* melanocytes (Fig. 4a), which, while not affecting the total number of pigmented melanosomes (Fig. 4b), resulted in the redistribution of the pigmented melanosomes in the perinuclear area (Fig. 4c, d).

### Decreased Dynlt3 amounts increased melanosome motility

We next examined potential alterations in melanosome motility using time-lapse video microscopy to track pigmented melanosome movement (Supplementary Videos 1 and 2). Direct observation of melanosome movements revealed that motion is generally composed of variable and intermittent movement, with a high level of pauses followed by short bursts of movements, as previously reported^39,40^.

**Fig. 5:**
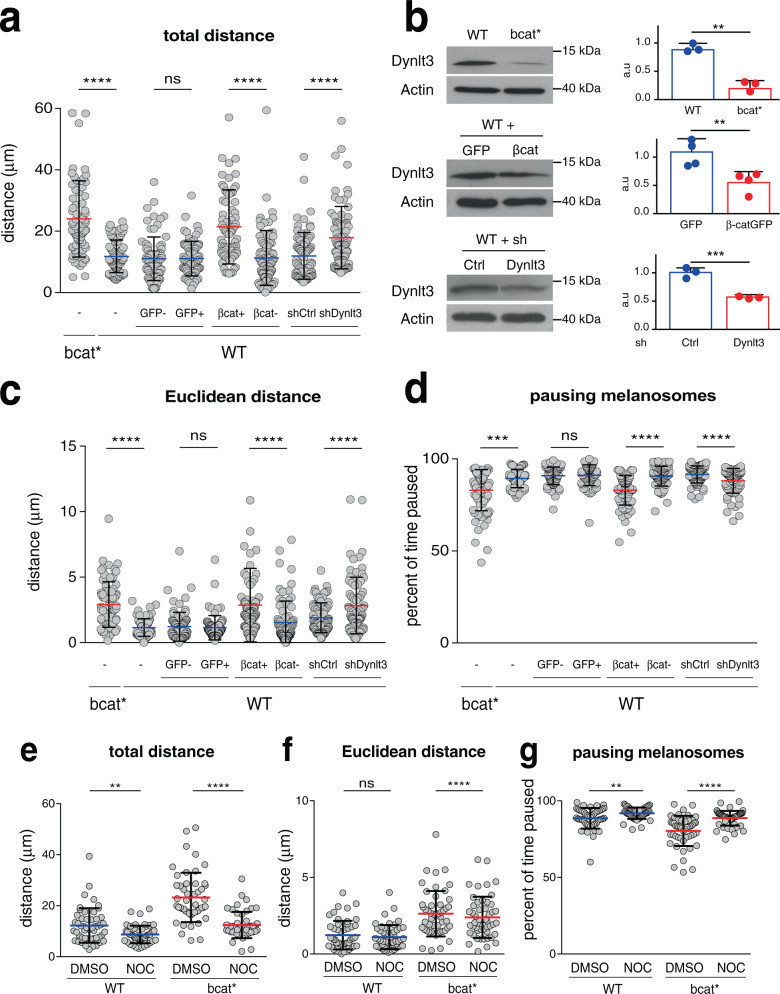
Melanosome movement is increased in cells producing active β-catenin or with diminished levels of Dynlt3. Melanosome movement (corresponding mainly to stage IV melanosome) was assessed by brightfield video microscopy over a period of 5 min and melanosome trajectories were followed using ImageJ software. For each cell line, 75 melanosomes were followed from a minimum of nine independent cells. WT cell lines were transfected with control GFP or β-catenin-GFP expression vectors, respectively. Analyses were performed on both transfected (i.e., GFP-positive) and non-transfected (i.e., GFP-negative) cells. In addition, WT cells were transfected with either control or Dynlt3 shRNA vectors, and analyses were performed on the resulting red cells. Each dot represents one melanosome. **a** The total distance indicates the sum of all of the individual tracks for each melanosome. **c** The Euclidean distance refers to the distance between the start and end position of each melanosome. **d** The percentage of time that each melanosome spent stationary/paused refers to the proportion of the number of tracks where no movement was measured compared to the total number of tracks. The pause depends on the time and the pixel size (1 pixel = 0.16 μm). **b** The level of Dynlt3 in these different cell lines was evaluated after western blot analysis. Relative quantification of the western blot analysis was performed from three to four independent biological experiments, with the mean ± SD presented. In panel **c**, one outlier was removed from WT+βcat+, its value was 20 μm. To assess the role of microtubules in melanosome movement, the total distance (**e**), Euclidean distance (**f**), and pausing melanosomes (**g**) were determined from 50 WT and bcat* melanosomes treated with 10 μM nocodazole (NOC) for 1 h. For western blot analysis, all statistical significance was determined using unpaired two-sided *t*-tests, from a minimum of three independent biological experiments. **p < 0.01; ***p = 0.0005. For the other analyses, statistical significance was measured using two-sided Mann–Whitney tests. ns signifies not significant (*p* = 0.4992 for all panels except panel **e**, where *p* = 0.5839), ****p < 0.0001, ***p = 0.0002, **p = 0.0046 (**e**) and 0.0053 (**g**).

The movement of melanosomes in bcat* cells was enhanced when compared with WT melanosomes. On average, the total distance that bcat* melanosomes traveled was longer than WT melanosomes (24.0 vs. 11.8 μm, Fig. 5a, b). The average distance and velocity of bcat* melanosomes were also increased compared to WT melanosomes (Supplementary Fig. 6a, b). Similarly, βcat-Δex3-GFP WT cells showed increased total distance traveled by melanosomes relative to WT cells expressing GFP alone (21.4 vs. 11.1 μm, Fig. 5a, b). The average distance and velocity of melanosomes in βcat-Δex3-GFP-expressing WT cells were also higher than in control GFP-expressing WT cells (Supplementary Fig. 6a, b). Additionally, following transfection with GFP or βcat-Δex3-GFP, we monitored the trajectories of melanosomes in non-transfected cells (i.e., GFP-negative cells). The total distance that the melanosomes traveled on average in these non-transfected cells (GFP or βcat-Δex3-GFP) was similar to WT cells (11.8 vs. 11.0 vs. 11.3 μm, respectively, Fig. 5a, b). Taken together, β-catenin exogenous expression increased the movement of melanosomes.

We also examined the directionality of the tracked melanosomes, with respect to the centrifugal and centripetal movement (Supplementary Fig. 6c, d). While melanosomes in WT cells moved centrifugally and centripetally with similar frequencies (51% centrifugal and 49% centripetal), significantly, bcat* melanosomes more frequently moved centrifugally (71% centrifugal vs. 29% centripetal). Similarly, βcat-Δex3-GFP WT melanosomes moved more centrifugally than centripetally (68% vs. 32%, respectively), whereas melanosomes in control GFP-transfected WT cells moved centrifugally and centripetally with similar frequencies (47% vs. 53%, respectively).

Downregulation of Dynlt3 in WT melanocytes stably expressing shRNA targeting this gene resulted in a significant decrease of Dynlt3 and an increase in the total distance melanosomes traveled compared to shCtrl cells (17.9 vs. 11.9 μm, Fig. 5a, b). This was accompanied by an increased average distance and average velocity of melanosomes (Supplementary Fig. 6a, b). Consistently, the Euclidean distance traveled by the melanosomes was increased after overexpression of β-catenin or downregulation of Dynlt3 (Fig. 5c).

In accordance, while melanosomes in WT-shCtrl cells moved centrifugally and centripetally with similar frequencies (54% centrifugal and 46% centripetal), melanosomes in WT-shDynlt3 cells more frequently moved centrifugally (69% centrifugal vs. 31% centripetal) (Supplementary Fig. 6c, d).

To further characterize the melanosome movement, the percentage of pausing melanosomes was determined by considering the number of frames where no movement was recorded. An observed pausing melanosome can be the result of movements below the resolution limit of the microscope, a biological lack of movement of the melanosome, or the equal, yet opposite tug-of-war action of opposing molecular motors, leading to a perceived lack of movement. Tracked melanosomes in bcat*, WT-βcat-Δex3 or WT-shDynlt3 cells paused less frequently than controls (Fig. 5b, d), showing together that Dynlt3, through βcat expression, contributes, at the very least, to the intracellular dynamics of pigmented melanosomes.

Finally, we assessed whether the increased movement of melanosomes in bcat* cells was microtubule-dependent by treating WT and bcat* cells with 10μM of nocodazole (NOC) for 1 h to depolymerize the microtubules and then assessed melanosome movement. Interestingly, treatment with nocodazole significantly decreased melanosome movement in bcat* cells, as both the total distance and the Euclidean distance were decreased compared to the DMSO control (Fig. 5e, f). Consistently, the percentage of paused melanosomes was increased following nocodazole treatment compared to the DMSO control (Fig. 5g). Nocodazole treatment also decreased the total distance melanosomes traveled and increased the percentage of paused melanosomes in WT cells, but not the Euclidean distance (Fig. 5e–g). However, the effects of nocodazole were more pronounced in bcat* cells compared to WT cells. Thus, the increased movement of melanosomes in bcat* cells appears to be dependent on microtubules.

### Mathematical and statistical analyses reveal an increase in directional melanosome movement after reduction of Dynlt3 levels

Displacement of an object can be generated by a superposition of various elementary processes: Brownian movement (diffusion) as a basic process, with the potential addition of directional movement, elastic tethering (flexible attachment to a fixed point), and/or confinement. Noise analysis (e.g., tracking error) also has to be taken into account for the analysis of experimental trajectories (see Supplementary Note 1). In the context of a general approach aimed to identify the elementary physical processes involved in melanosome movement^41^, we quantified the proportion of trajectories that were directional during the time interval of our observation.

**Fig. 6:**
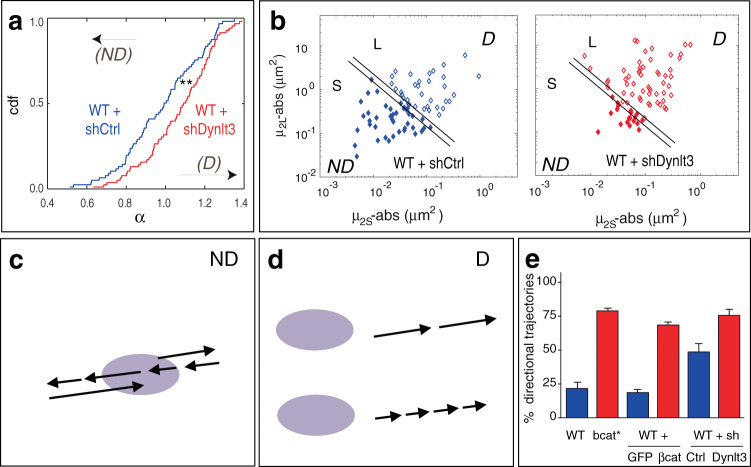
Increased directional movement of melanosomes in cells with increased levels of β-catenin or decreased Dynlt3. **a** *α* distributions for WT-shCtrl (blue) and WT-shDynlt3 (red) populations of trajectories. Non-directional (ND) and directional (D). Statistical analyses were done using a Wilcoxon test. **p < 0.01. cdf, cumulative distribution function. **b** μ_2S_, μ_2L_ plots reflecting the extent of WT-shCtrl (**b**) and WT-shDynlt3 (**c**) trajectories. Each point corresponds to one trajectory. Lines correspond to frontiers between ND and D trajectories: see Supplementary Note 2 for criteria and validation. **c**, **d** Schematic representation of ND (**c**) and D (**d**) movement. The purple ellipse represents the cargo. The arrows represent the direction and the orientation of the movement, and the length of the arrows is directly proportional to the magnitude of the movement. **e** Percentage of directional trajectories for melanosomes in WT, bcat*, WT-GFP (denoted WT + GFP), WT-βcat-Δex3-GFP (denoted WT + βcat), WT-shCtrl, and WT-shDynlt3 cells, out of the total 75 trajectories for each population of trajectories. Cell lines depicted in blue have trajectories that are mainly non-directional, and those in red are mainly directional. The values presented in the graph correspond to the middle values of the ranges described in the text. The same frontiers were used for all populations (Supplementary Note 2). Error bars correspond to trajectories between the two frontiers (Fig. 6b and Supplementary Fig. 7).

We focused on a descriptor referring to the classical analysis of the Mean-square displacement (${\mathrm{MSD}}(\tau ) = < [r(t + \tau ) - r(t)]^2 >$), where *r*(*t*) is the position at a given time *t* and *τ* is the time interval used to calculate the displacement. Here, the MSD was fit with *bτ*^*α*^ and *α*, the MSD exponent, was taken as our main descriptor (Fig. 6a, *α* cumulative distribution function, cdf). In the case of purely 2D diffusive motion, ${\mathrm{MSD}}(\tau ) = 4D\tau$ (*D* diffusion coefficient, ideal case without analysis noise), while for purely directional movement, ${\mathrm{MSD}}(\tau ) = V^2\tau ^2$ (*V* velocity). We previously studied the distribution of *α* for different simple generating processes^41^ and as expected, the α distribution was centered around 1 for diffusive processes. However, in cells, the likelihood that organelles move in a diffusive manner is low. For *α* < 1, the movement could be considered as tethering or confined processes. Such processes were widely reported for organelle movements due to transient interactions, confinement, or cytoplasmic crowding^42–44^, but also for the addition of a Gaussian noise^41^, corresponding, for example, to tracking or analysis noise^45,46^. For *α* > 1, the movement is directional and it is important to note that the higher the value of α (when *α* > 1), the more the movement is directional. Here, this analysis was used to bring to light differences in the proportion of the trajectories that showed significant directional movement within the time scale of our observation (5 min). Analysis of the α distributions in WT-shDynlt3 and WT-shCtrl cells showed melanosome movement was more directional in cells with decreased Dynlt3 levels, as the α distribution was shifted significantly to the right in WT-shDynlt3 cells compared to WT-shCtrl cells (Fig. 6a). Similarly, there was a statistically significant difference between the α distributions of WT and bcat* cells and between WT-GFP and WT- βcat-Δex3-GFP cells (Supplementary Fig. 7), with the melanosomes in cells overexpressing β-catenin displaying more directional movement.

To further characterize the movement of melanosomes, we used a second descriptor, the second moments of trajectories, to generate small [S] and large [L] amplitude subpopulations (Fig. 6b). To do so, we used Brownian movement as a mathematical reference, which is shown to enhance the visualization of right- and left-shifted distributions corresponding to these directional and non-directional trajectories, respectively^41^. The frontier was chosen such that L mainly corresponded to a homogeneous, directional (D) population, and S to a non-directional (ND) population (Fig. 6c, d and Supplementary Notes 1, 2), with trajectories between the two lines defining an error bar. The number of directional trajectories (out of 75 trajectories) was estimated to be 13–19 (17–25%) for WT, compared to 57–61 (76–81%) for bcat*; 12–16 (16–21%) for WT-GFP compared to 50–52 (67–69%) for WT-βcat-Δex3-GFP (Supplementary Fig. 7); and 32–41 (43–55%) for WT-shCtrl compared to 53–61 (71–81%) for WT-shDynlt3 (Fig. 6e). This illustrates that the downregulation of Dynlt3 or the expression of active β-catenin both leads to a significant increase of directional trajectories.

### Overexpression of β-catenin or reduction of Dynlt3 diminishes melanosome deacidification and transfer

**Fig. 7:**
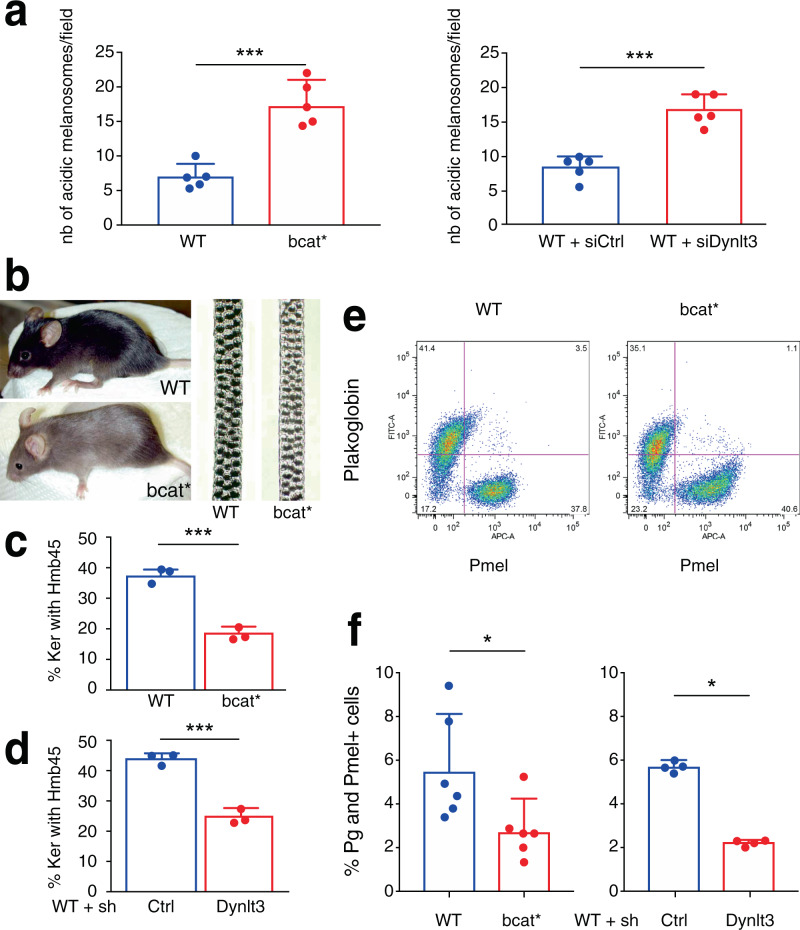
Melanosome acidity and transfer are altered in cells producing active β-catenin or with a diminished level of Dynlt3. **a** Quantitation of acridine orange positive melanosomes in WT, bcat*, WT + siCtrl, and WT + siDynlt3 cells from five independent biological experiments. ***p = 0.0007 (WT vs. bcat*) and 0.0001 (WT + siCtrl vs. WT + siDynlt3), as determined by unpaired, two-sided *t*-tests. Nb, number. **b** Photograph of wild-type and bcat* 2-month-old adult male mice and of their respective dorsal hairs. **c** Quantitation of the percentage of Pmel and Plakoglobin double-positive cells in co-cultures of WT or bcat* cells with Balb/c MK keratinocytes, as assessed by confocal microscopy. Pmel positivity was assessed as being either positive or negative within each respective keratinocyte (Ker). Quantitation was done from three biologically independent co-culture experiments. ***p = 0.0004, as determined by an unpaired, two-sided *t*-test. **d** Quantitation of the percentage of Pmel and Plakoglobin double-positive cells, in co-cultures of WT cells expressing either control or Dynlt3 shRNA, with Balb/c MK keratinocytes, as assessed by confocal microscopy. Pmel positivity was assessed as being either positive or negative within each respective keratinocyte (Ker). Quantitation was done from three biologically independent co-culture experiments. ***p = 0.0005, as determined by an unpaired, two-sided *t*-test. **e** FACS analysis of Hmb45 and plakoglobin positive cell populations in WT and bcat* melanocytes. **f** Quantitation of melanosome transfer assayed by FACS. WT, bcat*, WT-shCtrl, or WT-shDynlt3 melanocytes were co-cultured with Balb/c MK mouse keratinocytes for 10 days, after which the co-cultures were fixed and processed for FACS analyses using Hmb45 and Plakoglobin (Pg) antibodies. The percentage of Plakoglobin and Hmb45 double-positive cells within each respective co-culture is presented. Quantitation was done from a minimum of four biologically independent co-culture experiments. For all quantitations, the mean ± SD is shown. *p = 0.0260 (WT vs. bcat*) and 0.0286 (WT-shCtrl vs. WT-shDynlt3), as determined by an unpaired, two-sided *t*-test.

Melanosome acidity, which is one indication of melanosome maturation, was evaluated in cells expressing different amounts of Dynlt3. Non-fully mature melanosomes are more acidic than mature melanosomes. In this context, the number of acidic and pigmented melanosomes was approximately two-fold higher in bcat* cells relative to WT cells (17.2 vs. 7.0 acidic melanosomes/field), as well as in WT-siDynlt3 vs. WT-siCtrl (16.8 vs. 8.4) (Fig. 7a and Supplementary Fig. 8).

Melanocytes with increased β-catenin or decreased Dynlt3 levels harbored more pigmented and peripheral melanosomes, which would represent an ideal subpopulation of pigment granules to be transferred to keratinocytes. However, their change in pH might reflect a failure to acquire transfer capacity^5^. We thus examined whether these cells would have a deficiency in melanosome transfer and consequently on coat color. Indeed, the hairs of bcat* C57BL/6J mice were lighter than WT C57BL/6J mice (Fig. 7b). Melanosome transfer was evaluated by co-culturing WT, bcat*, WT-shCtrl, and WT-shDynlt3 melanocytes in the presence of Balb/c MK keratinocytes for 10 days prior to immunofluorescence analysis using plakoglobin (keratinocyte marker) and Hmb45 (a melanosome marker labeling the intraluminal melanin-positive fibrils) (Supplementary Fig. 9a, b). We then counted the number of keratinocytes in which we could detect at least one Hmb45-positive structure; this number was significantly lower when Dynlt3 was lower than normal (Fig. 7c, d). In addition, we further confirmed that melanosome transfer was decreased in bcat* and shDynlt3 melanocytes after FACS analysis (Fig. 7e, f and Supplementary Fig. 9c).

Taken together, these data suggest that in melanocytes with stable, activated β-catenin or in those with diminished expression of Dynlt3, both melanosome acidity and transfer are negatively affected.

## Discussion

In this study, we showed that in melanocytes derived from transgenic mice expressing active β-catenin (bcat* cells), pigmented melanosomes were primarily absent from the perinuclear area and localized at the cell periphery. Notably, the expression of Dynlt3, a member of the cytoplasmic dynein complex, was downregulated in bcat* cells, and knockdown of Dynlt3 in WT melanocytes phenocopied the peripheral localization of pigmented melanosomes and their absence from the perinuclear area. Melanosome movement was increased and melanosomes moved in a more directional manner in cells with diminished levels of Dynlt3. Decreased levels of Dynlt3 resulted in more acidic melanosomes that were associated with a reduction of melanosome transfer to keratinocytes. Altogether, these results show that Dynlt3 is a key player in regulating melanosome movement, acidity, and transfer. As a target of β-catenin, our results reveal a function for the Wnt/β-catenin signaling pathway during melanocyte and skin pigmentation.

Dynlt3 is a component of the cytoplasmic dynein complex, a macromolecular protein complex that is essential for the transport of cargos from the cell periphery towards the nucleus^17,38^. While the heavy chain (Dync1h1) of this complex has an ATPase activity and interacts with the microtubules, the light intermediate (Dync1li1 and Dync1li2) and the light chains, including Dynlt3, along with dynactin, a dynein-interacting cofactor, and activator, are thought to have a role in cargo recognition. Despite previous studies have shown that various virus capsid proteins (e.g., papilloma and herpes) interact with Dynlt3 and hijack the Cytoplasmic Dynein complex to reach the nucleus of infected cells^47,48^, this work is the first to functionally demonstrate that Dynlt3 is involved in the intracellular trafficking of an organelle.

To date, transgenic mice with melanocyte-specific mutations in cytoplasmic dyneins have neither been identified nor produced and therefore the role of Dynlt3 in mouse pigmentation remains unknown. However, transgenic mice containing heterozygous mutations in Dync1h1, which encodes the dynein heavy chain Dync1h1, have been produced. In general, these mice show defects in motor functions, with motor neuron degeneration and/or sensory neuropathy observed^49–52^. Yet, no obvious coat color phenotypes were reported in these heterozygous mice. Importantly, homozygous Dync1h1 mice are not viable and die during embryonic development or shortly after birth. Therefore, whether a coat color phenotype would be observable in homozygous mice but would not be apparent in heterozygous mice remains unknown. Conditional mouse mutants would be required to answer this point, using for instance the Tyr::Cre mice^53^.

While mutations in DYNLT3 are not significantly detailed in human disease, reduction of DYNLT3 protein has been observed in late-stage Parkinson’s disease; however, whether or not decreased levels of DYNLT3 protein have a role in Parkinson’s disease or whether they are simply the result of the disease’s progression remains unclear^54^. Interestingly, recent epidemiological studies have suggested that patients with Parkinson’s disease have a higher risk of developing melanoma, and vice versa^55^. PARKIN, which is frequently mutated in young-onset Parkinson’s disease, is also downregulated in melanoma, providing a molecular link between these two diseases^56^. However, to the best of our knowledge, an association between DYNLT3 and melanoma has not been demonstrated. Further studies will have to be performed to evaluate whether DYNLT3 protein levels are decreased in melanoma, at least in the context of melanoma patients with Parkinson’s. Intriguingly, a recent study demonstrated that the levels of DYNLT3 protein were elevated in ovarian epithelial lesions and that high levels of DYNLT3 mRNA were related to decreased overall survival and progression-free survival^57^.

Microarray analyses of LRO trafficking genes demonstrated that among the components associated with cytoplasmic dynein, only Dynlt3 levels were altered in response to β-catenin expression, suggesting that only Dynlt3 is a target of β-catenin. Interestingly, the *Dynlt3* promoter contains four putative Tcf/Lef binding sites, suggesting that β-catenin may be involved in the regulation of Dynlt3 transcription. Importantly, our results identify Dynlt3 as a β-catenin target and one whose expression is negatively regulated by β-catenin. However, we cannot exclude that knockdown of other dynein complex members may have a similar effect on melanosome localization. Importantly, in WT and bcat* cells, Dynlt1, the other member of the Tctex light chain family, was not expressed (Supplementary Fig. 5b), in agreement with previous studies showing that Dynlt1 and Dynlt3 are not expressed in the same tissue^24,25^. Whether other cytoplasmic dynein subunits affect melanosome localization, and which ones, in particular, do so, are currently under investigation.

Video microscopy and manual tracking showed that melanosome movement is not constantly directional. Instead, the direction that melanosomes moved changed consistently, with melanosomes often going forward and backward repeatedly. Melanosome movement in both WT and bcat* cells was significantly inhibited following treatment with nocodazole, which was consistent with previous reports showing decreased melanosome motility in *Xenopus* melanophores and mouse RPE cultures following nocodazole treatment^9,40,58–61^.

The downregulation of Dynlt3 resulted in a more directed movement. On microtubules, the back and forth movement of melanosomes can be thought of as competitions between the kinesin and dynein motors. While this random movement would be energetically costly and seemingly inefficient^62^, this back and forth movement may occur to allow melanosomes to mature properly without passing onto the actin microfilaments and being transferred before they are fully mature. Furthermore, these types of melanosome saltations in different directions, which are characterized by rapid bursts of movement interspersed with pauses, are thought to be important for the proper homogenous distribution of melanosomes in the cytoplasm and could be useful for helping them avoid potential traffic jams on microtubules^9,58,63^.

To our knowledge, this work is the first indication that defects in cytoplasmic dynein have an impact on melanosome transfer. That decreased Dynlt3 levels diminished melanosome transfer is somewhat perplexing since pigmented melanosomes localized primarily at the cell periphery, and were ideally positioned to be further transferred. However, in cells that expressed less Dynlt3, melanosomes were more acidic, as more pigmented melanosomes accumulated acridine orange. In addition, in bcat* cells, melanosomes were larger and more acidic than WT melanosomes. This is coherent and we hypothesize that this occurs since the bcat* melanosomes were not able to de-acidy their lumens and remove the materials which are supposed to remain in melanocytes before the mature melanosomes are transferred to keratinocytes, including melanogenic enzymes such as tyrosinase and Tyrp1. Since melanosomes de-acidify their lumen before being transferred to keratinocytes, and since increased acidity is an indication of immature melanosomes, our data suggest that Dynlt3 has some role in the regulation of melanosome maturity, or at the very least, in the deacidification of the melanosome lumen. Recently, it has been shown that the maturation of melanosomes before transfer involves the formation and release of membrane tubules containing proteins (e.g., Vamp7) that are needed to tune melanosome homeostasis (including the pH and melanin content) and ultimately their secretory capacity^5^. The separation of these tubules from the mature melanosome requires proteins involved in membrane scission (e.g., Myosin 6, Optineurin), whose downregulation results in increased melanosome size and acidity. One should note that Myosin 6 was upregulated in bcat* vs. WT melanocytes (Fig. 2a). Our hypothesis is that since the level of Dynlt3 is reduced, the cell tries to compensate for this reduction by increasing the amount of Myosin 6 to allow the separation of these tubules from the melanosomes. Therefore, it is conceivable that Dynlt3 has a role in this process, possibly in the pulling, fission, and/or the retrograde transport of the melanosomal tubules, therefore contributing to melanosome maturation and secretion.

Taken together, our results have, to the best of our knowledge, identified Dynlt3 as a novel and important player in melanosome biology (transport, acidity/maturation, and transfer) and in coat coloration and pigmentation. Furthermore, our results suggest that cytoplasmic dynein proteins may have other roles in the biology of different organelles, in addition to their already established functions in organelle trafficking.

## Methods

### Cell culture

Immortalized mouse melanocyte cell lines established from the skin of wild-type (WT) and bcat* *Mus musculus* C57BL/6J male transgenic mice have been previously described^34^. Melanocyte cell lines were grown in Ham’s F12 media (Gibco, 21765-029) supplemented with 10% FBS (Gibco, 10270-106), antibiotics (100 U/mL penicillin and 100 μg/mL streptomycin; Gibco, 15140), and 200 nM TPA (Sigma, P1585). Two independent wild-type (9v and 13d) and two independent bcat* (10d and 14d) melanocyte cell lines were used for this study. The melanocyte cell cultures contained only melanocytes, as keratinocytes do not grow under these media conditions and fibroblasts were selected against using the classical G418 protocol during cell line establishment. Balb/c MK mouse epidermal keratinocytes (obtained from ATCC) were grown in Joklik Modified MEM (Sigma, M8028) supplemented with 10% FBS, antibiotics (as above), and 5 ng/mL EGF (Sigma, E9644). All cell lines were maintained at 37 °C in a humidified atmosphere containing 5% CO_2_. All cell lines were routinely tested for mycoplasma and were all negative.

For co-culture experiments, the melanocyte and keratinocyte cell lines were cultured in melanocyte growth media in the presence of 200 nM TPA, 5 ng/mL EGF, and 50 nM CaCl_2_. Co-culture experiments were performed with a ratio of 2:1, keratinocytes to melanocytes, and the cells were left in co-culture for 10 days, after which they were processed as described below. For immunofluorescence experiments, the cells were plated on glass coverslips coated with 1 mg/mL collagen (Sigma, C7661).

Animal care, use, and experimental procedures were conducted in accordance with recommendations of the European Community (86/609/EEC) and Union (2010/63/UE), and the French National Committee (87/848). Animal care and use were approved by the ethics committee of the Curie Institute in compliance with the institutional guidelines.

### Transfection and infection of melanocyte cell lines

All cells were transfected using the Amaxa Cell Line Nucleofector Kit L (Lonza, VCA-1005) according to the manufacturer’s recommendations. Briefly, cells were trypsinized and counted, with 7.5 × 10^5^ cells resuspended in the Amaxa transfection reagent. Four micrograms of each respective plasmid or 100 pmol of siRNA were added to the mixture, which was then transferred to a cuvette and incubated in the Amaxa nucleofector. Following nucleofection, the cells were added to a six-well dish containing pre-warmed growth media. The following day, the cells were washed and incubated in fresh growth media supplemented with TPA. Transfections were assayed 48 h following nucleofection. Control GFP and β-catenin-GFP-tagged plasmids have been previously described^37^. The Dynlt3-GFP (pCMV3-mDynlt3-GFPSpark) and the control GFP vectors (pCMV3-GFP) were purchased from Sino Biological (MG5A2492-ACG and CV026, respectively).

WT cells expressing a control shRNA or an shRNA against Dynlt3 (targeting sequence: 5′-ATCAGATGTTGTATCCCAA-3′) were produced using viral particles and Dharmacon SMARTvector plasmids (VSC11715 and V3SM11241-233912870, respectively). Following infection, cells were selected using 1 μg/mL puromycin.

### siRNA sequences

siRNA targeting mouse *β-catenin* and *Dynlt3* were purchased from Dharmacon as a SMARTpool mix of 4 sequences. The sequences of the siRNA are as follows: for *β-catenin*: 5′-GAACGCAGCAGCAGUUUGU-3′, 5′-CAGCUGGCCUGGUUUGAUA-3′, 5′-GCAAGUAGCUGAUAUUGAC-3′, 5′-GAUCUUAGCUUAUGGCAAU-3′; and for Dynlt3: 5′-CCCAUAAUAUAGUCAAAGA-3′, 5′-GGUGGUAACGAUUAUAAUG-3′, 5′-GGGGAAAGCUUACAAGUAC-3′, 5′-CAGAGGAGCCCGUAUGGAU-3′. A random, scrambled sequence (5′-AAUUCUCCGAACGUGUCACGU-3′) was used as a control.

### Semi-quantitative real-time PCR

Total RNA was extracted from cells using the miRNeasy kit from Qiagen (217004) according to the manufacturer’s protocol. Reverse transcription reactions were performed with 500 ng of total RNA and M-MLV reverse transcriptase (Invitrogen), according to the manufacturer’s instructions. The cDNA generated was then subjected to semi-quantitative real-time PCR for the analysis of gene expression using ABI 7900HT. Oligonucleotides used for PCR were as follows: for *β-catenin*, 5′-CGTGGACAATGGCTACTCAA-3′ (F) and 5′-TGTCAGCTCAGGAATTGCAC-3′ (R); for *Dynlt3* 5′-GCGATGAGGTTGGCTTCAATGCTG-3′ (F) and 5′-CACTGCACAGGTCACAATGTACTTG-3′ (R); and for *Gapdh* 5′-ACCCAGAAGACTGTGGATGG-3′ (F) and 5′-CACATTGGGGGTAGGAACAC-3′ (R). F means forward. R means reverse.

### Western blot analysis

Whole-cell lysates were prepared from melanocyte cell lines using ice-cold RIPA buffer supplemented with a complete protease inhibitor cocktail and PhosStop phosphatase inhibitor cocktail (Roche). For western blotting, 25 μg total protein was separated on 15% denaturing acrylamide SDS-PAGE gels and the proteins transferred to nitrocellulose membranes. Membranes were blocked in 5% non-fat milk in Tris-buffered saline supplemented with 0.05% Tween-20 (TBST) and probed with primary antibodies overnight. The signal was detected using peroxidase-conjugated anti-mouse or anti-rabbit secondary antibodies (Jackson, 115-035-003 and 111-035-003, respectively, both used at 1:20,000) and enhanced chemiluminescence (ECL; ThermoFisher). The primary antibodies were used as follows: Dynlt3, 1:200 (Sigma, hpa003938), β-catenin, 1:2000 (Abcam, ab6302), GFP, 1:500 (Institut Curie), and β-actin, 1:10,000 (Sigma, A5441).

### Immunofluorescence microscopy

Melanocyte cells were seeded on 18 mm glass coverslips and grown to confluence, after which they were washed twice with ice-cold PBS and fixed-permeabilized with ice-cold methanol–acetone (1:1 solution) on ice for 5 min. Next, cells were washed twice with cold PBS and blocked with a blocking solution containing 1% BSA (w/v) and 10% FBS in PBS for 1 h at room temperature. Cells were washed again twice with PBS and then incubated with primary antibodies against anti-Tyrp1 (Abcam, ab3312) and anti-GFP (Institut Curie) both at a concentration of 1:200, at 4 °C overnight. The following day, the cells were washed again three times with PBS and then incubated with Alexa 555 anti-rabbit (ThermoFisher, A231572) or Alexa 488 anti-mouse (ThermoFisher, A21202) secondary antibodies (1:500 for both) for 1 h at room temperature in the absence of light. All antibodies were diluted in PBS + 1% BSA. Cells were then again washed two times before being counterstained with 0.5 µg/µL DAPI in PBS to visualize the nucleus. Coverslips were then mounted on glass slides using ProLong Gold antifade reagent (Invitrogen, P36934). Images were then taken using an Upright widefield Leica Microscope at ×63.

For chemical inhibitor experiments, WT cells were treated with 80 μM ciliobrevin D (Sigma, 250401) for 6 h. Cells were then washed twice with ice-cold PBS and fixed-permeabilized with ice-cold methanol–acetone (1:1 solution) on ice for 5 min. The cells were then stained for DAPI and mounted on coverslips as described above. Images were then taken using an Upright widefield Leica DM6000 Microscope equipped with a ×63 oil-immersion objective. Bloc filters were used to detect fluorescence: for DAPI, excitation was performed between 375 and 435 nm, and emission was collected between 445 and 495 nm, for Alexa 488, excitation was between 450 and 490 nm and emission was collected between 500 and 550 nm, and Alexa 555, excitation was between 590 and 650 nm and emission was collected between 663 and 738 nm. Images were obtained with an sCMOS Hamamatsu Orca Flash camera.

For co-culture experiments, mouse-keratinocyte co-cultures were washed twice with cold PBS and then fixed with 4% paraformaldehyde (VWR 1.04005.1000) for 20 min at room temperature. Following two, 5-min washes with PBS, the cells were permeabilized with 0.2% v/v Triton X-100 in PBS for 10 min at room temperature. The cells were once again washed and then processed as described above, using anti-Hmb45 (Abcam, 787) and anti-plakoglobin (Abcam, 184919) primary antibodies both diluted to 1:200.

Confocal images of co-cultures were acquired with a Leica SP5 inverted confocal laser scanning microscope (CLSM), equipped with a ×63 Plan Apochromat oil‐immersion objective. The pinhole was set to 1 AU. No zoom was used during acquisition for co-culture experiments whereas a zoom of ×3.5 was used for acridine orange experiments. Fluorescence emissions were detected at 575–645 nm upon excitation at 561 nm (DPSS laser) for Alexa 555, at 500–550 nm upon excitation at 488 nm (Argon laser) for Alexa 488, and at 415–465 nm upon excitation at 405 nm (Diode laser) for DAPI. Images were obtained with a PMT detector.

### Video microscopy and melanosome tracking

Cells were seeded in 6-well glass plates (MatTek Corporation P06G-1.5-20-F) and were imaged using a Leica DMI6000B inverted widefield microscope (Ratio 2) equipped with an EM-CCD Photometrics camera. Cells were maintained at 37 °C in a humidified atmosphere of 5% CO_2_ while videos were being taken. Videos were taken with a ×100 oil-immersion objective over a period of 5 min, with one image taken every 0.5 s. Videos were taken from a minimum of nine melanocytes per cell line/transfection from three independent biological experiments. From these melanocytes, the trajectories of 75 pigmented melanosomes were manually followed using the Manual Tracking plugin for ImageJ^64^. Each melanosome path was chosen at random. The Manual Tracking provided a number of different parameters that were used to characterize the movement of the individual melanosomes. Namely, we calculated the total distance the melanosomes moved, which was the sum of values for all of the 601 frames during the 5-min videos. Note that the distance traveled is dependent on the used time window. The Euclidean distance was calculated as the distance between the melanosome position at the first frame and the last frame^65^. The average distance was calculated as the total distance divided by the number of frames (601). Similarly, the average velocity was calculated by taking the average distance per frame and dividing it by the time (0.5 s). Finally, the percentage of time the melanosomes were paused was calculated by assessing the number of frames (out of the 601 total frames) where a “0” value was recorded for the distance traveled. Using the Ratio 2 video microscope, the size of each pixel was 0.16 μm, and therefore, in our analyses, movements below this size were considered to be static. Therefore, an observed paused melanosome can be the result of movements below the resolution limit of the microscope, a biological lack of movement of the melanosome or the equal, yet opposite tug-of-war action of opposing molecular motors, leading to a perceived lack of movement. The distribution of spatiotemporal descriptors of trajectories, in particular mean-square displacement characteristics, was computed and compared to simulated trajectories in order to distinguish directional *vs*. non-directional movements (ref. ^41^ and Supplementary Notes 1 and 2). Tracking was done blindly by two different individuals.

For video microscopy following nocodazole treatment, WT and bcat* cells were incubated in 10 μM nocodazole (Sigma, M1404) for 1 h at 37 °C. Videos were then taken in a humidified atmosphere of 5% CO_2_ at 37 °C.

### Melanosome number and localization quantification

Brightfield photomicrographs of melanocytes were obtained with an upright widefield Leica DM6000 microscope. Using the default threshold of the ImageJ Analyze Particle feature, we selected pigmented structures with a size greater than or equal to 150 nm in order to estimate the apparent number of pigmented melanosomes/cell. Albino melanocyte melan-c was used as a non-pigmented reference. To examine perinuclear melanosomes, we used a DAPI image of each cell of interest to establish the perinuclear area. Specifically, using the edge of the nucleus as a starting point, we considered an area that was 2 μm extended from the border of the nucleus. The number of perinuclear melanosomes in this “nuclear-extended” boundary region was then counted and normalized to the total area of the boundary region. In the case where some of the regions extended outside of the cell, this area was subtracted from the area used for quantification. Quantification of melanosome numbers and localization was done blindly by two different individuals.

### Flow cytometry

For all flow cytometry experiments, cells were trypsinized and the resulting cell pellets were resuspended and fixed in 4% paraformaldehyde for 20 min at room temperature. Following centrifugation, the cell pellets were then washed twice with PBS containing 1% BSA and 0.1% saponin (Sigma, S4521). Cells were then incubated in primary antibodies against Hmb45 and plakoglobin (both at 1:100) for 2 h at 4 °C while rocking in the absence of light. Next, cell pellets were washed once with PBS/BSA/saponin and incubated in Alexa 647 rabbit (ThermoFisher A31573) and Alexa 488 mouse secondary antibodies (at 1:500) for 1 h. Cells were then washed in PBS and resuspended in PBS + 0.1% azide. Flow cytometric analyses were performed using a BD FACSCanto II machine.

### Melanosome acidity

Cells were seeded in 35 mm imaging dishes (Ibidi, 81156) and allowed to reach ~75% confluence. Acridine orange (Sigma, A6014) was added to the cells at 20 μg/mL and incubated for 20 min at 37 °C. The cells were then incubated in warm PBS and imaged using a confocal microscope. To look at acridine orange staining, fluorescence emissions were detected at 590–660 nm upon excitation at 458 nm (Argon laser)^66^. Melanosome acidity was assessed by counting the number of pigmented melanosomes that were positive for acridine orange staining. This was done by merging the brightfield and acridine orange signals. The number of melanocytes assessed per field of view was always kept constant.

### Electron microscopy

Melanocytes seeded on coverslips were chemically fixed in 2.5% (v/v) glutaraldehyde, 2% (v/v) paraformaldehyde in 0.1 M cacodylate buffer for 24 h at 4 °C, post-fixed with 1% (w/v) osmium tetroxide supplemented with 1.5% (w/v) potassium ferrocyanide, dehydrated in ethanol and embedded in Epon^67^. Ultrathin sections of cell monolayers or tissue were prepared with a Reichert UltracutS ultramicrotome (Leica Microsystems) and contrasted with uranyl acetate and lead citrate. Electron micrographs were acquired on a Tecnai Spirit electron microscope (FEI, Eindhoven, The Netherlands) equipped with a 4k CCD camera (EMSIS GmbH, Münster, Germany). The area of the melanosomes was determined as the length * width * π. Measurements were done blindly by two different individuals.

### Statistics and reproducibility

The details of statistical tests and the number of replicates for each experiment are provided in the figure legends and in the “Methods” section. The data were analyzed using GraphPad Prism. The values in the graphs represent the mean of at least three independent biological experiments, with error bars representing the standard deviation (SD). Any *p*-value inferior to 0.05 was considered to be statistically significant.

### Reporting summary

Further information on research design is available in the Nature Research Reporting Summary linked to this article.

## Supplementary information

Supplementary Information

Description of Supplementary Files

Supplementary Movie 1

Supplementary Movie 2

Supplementary Data 1

Supplementary Data 2

Reporting Summary

## Data Availability

The Affymetrix transcriptomic data generated during this study have been deposited in the NCBI GEO and are available under the accession number GSE167268. All numerical source data for the main figures are included in this published article’s Supplementary Data 2 file.

## References

[1] Colombo, S., Berlin, I., Delmas, V. & Larue, L. in *Melanins and melanosomes* (eds Riley, P. A. & J. Borovansky, J.) 21–51 (2011).

[2] Delevoye C, Marks MS, Raposo G (2019). Lysosome-related organelles as functional adaptations of the endolysosomal system. Curr. Opin. Cell Biol..

[3] D’Alba L, Shawkey MD (2019). Melanosomes: biogenesis, properties, and evolution of an ancient organelle. Physiol. Rev..

[4] Tarafder AK (2014). Rab11b mediates melanin transfer between donor melanocytes and acceptor keratinocytes via coupled exo/endocytosis. J. Investig. Dermatol..

[5] Ripoll L (2018). Myosin VI and branched actin filaments mediate membrane constriction and fission of melanosomal tubule carriers. J. Cell Biol..

[6] Dennis MK (2016). BLOC-1 and BLOC-3 regulate VAMP7 cycling to and from melanosomes via distinct tubular transport carriers. J. Cell Biol..

[7] Hume AN, Seabra MC (2011). Melanosomes on the move: a model to understand organelle dynamics. Biochem. Soc. Trans..

[8] Jiang, M. *et al*. Microtubule motor transport in the delivery of melanosomes to the actin-rich apical domain of the retinal pigment epithelium. *J. Cell. Sci*. **133**, 10.1242/jcs.242214 (2020). 10.1242/jcs.242214 PMC7420818 32661088

[9] Tuma MC, Zill A, Le Bot N, Vernos I, Gelfand V (1998). Heterotrimeric kinesin II is the microtubule motor protein responsible for pigment dispersion in *Xenopus* melanophores. J. Cell Biol..

[10] Hirokawa N, Noda Y, Tanaka Y, Niwa S (2009). Kinesin superfamily motor proteins and intracellular transport. Nat. Rev. Mol. Cell Biol..

[11] Pastural E (1997). Griscelli disease maps to chromosome 15q21 and is associated with mutations in the myosin-Va gene. Nat. Genet..

[12] Menasche G (2000). Mutations in RAB27A cause Griscelli syndrome associated with haemophagocytic syndrome. Nat. Genet..

[13] Menasche G (2003). Griscelli syndrome restricted to hypopigmentation results from a melanophilin defect (GS3) or a MYO5A F-exon deletion (GS1). J. Clin. Invest..

[14] Mercer JA, Seperack PK, Strobel MC, Copeland NG, Jenkins NA (1991). Novel myosin heavy chain encoded by murine dilute coat colour locus. Nature.

[15] Wilson SM (2000). A mutation in Rab27a causes the vesicle transport defects observed in ashen mice. Proc. Natl Acad. Sci. USA.

[16] Matesic LE (2001). Mutations in Mlph, encoding a member of the Rab effector family, cause the melanosome transport defects observed in leaden mice. Proc. Natl Acad. Sci. USA.

[17] Reck-Peterson SL, Redwine WB, Vale RD, Carter AP (2018). The cytoplasmic dynein transport machinery and its many cargoes. Nat. Rev. Mol. Cell Biol..

[18] Canty JT, Yildiz A (2020). Activation and regulation of cytoplasmic dynein. Trends Biochem. Sci..

[19] Pfister KK (2015). Distinct functional roles of cytoplasmic dynein defined by the intermediate chain isoforms. Exp. Cell Res..

[20] Pfister KK (2006). Genetic analysis of the cytoplasmic dynein subunit families. PLoS Genet..

[21] Vancoillie G (2000). Cytoplasmic dynein colocalizes with melanosomes in normal human melanocytes. Br. J. Dermatol.

[22] Byers HR, Yaar M, Eller MS, Jalbert NL, Gilchrest BA (2000). Role of cytoplasmic dynein in melanosome transport in human melanocytes. J. Investig. Dermatol..

[23] Reilein AR (2003). Differential regulation of dynein-driven melanosome movement. Biochem. Biophys. Res. Commun..

[24] King SM (1998). Cytoplasmic dynein contains a family of differentially expressed light chains. Biochemistry.

[25] Chuang JZ, Milner TA, Sung CH (2001). Subunit heterogeneity of cytoplasmic dynein: Differential expression of 14 kDa dynein light chains in rat hippocampus. J. Neurosci..

[26] Douglas MW (2004). Herpes simplex virus type 1 capsid protein VP26 interacts with dynein light chains RP3 and Tctex1 and plays a role in retrograde cellular transport. J. Biol. Chem..

[27] Lo KW, Kogoy JM, Pfister KK (2007). The DYNLT3 light chain directly links cytoplasmic dynein to a spindle checkpoint protein, Bub3. J. Biol. Chem..

[28] Yeh TY, Chuang JZ, Sung CH (2005). Dynein light chain rp3 acts as a nuclear matrix-associated transcriptional modulator in a dynein-independent pathway. J. Cell Sci..

[29] Hartsock A, Nelson WJ (2008). Adherens and tight junctions: structure, function and connections to the actin cytoskeleton. Biochim. Biophys. Acta.

[30] Aktary Z, Bertrand JU, Larue L (2016). The WNT-less wonder: WNT-independent beta-catenin signaling. Pigment Cell Melanoma Res..

[31] Takeda K (2000). Induction of melanocyte-specific microphthalmia-associated transcription factor by Wnt-3a. J. Biol. Chem..

[32] Goodall J (2004). Brn-2 expression controls melanoma proliferation and is directly regulated by beta-catenin. Mol. Cell. Biol..

[33] Lamoreux, M. L., Delmas, V., Larue, L. & Bennett, D. *The Colors of Mice: A Model Genetic Network* 297 (2010).

[34] Delmas V (2007). Beta-catenin induces immortalization of melanocytes by suppressing p16INK4a expression and cooperates with N-Ras in melanoma development. Genes Dev..

[35] Luciani F (2011). Biological and mathematical modeling of melanocyte development. Development.

[36] Raposo G, Tenza D, Murphy DM, Berson JF, Marks MS (2001). Distinct protein sorting and localization to premelanosomes, melanosomes, and lysosomes in pigmented melanocytic cells. J. Cell Biol..

[37] Yajima I (2013). A subpopulation of smooth muscle cells, derived from melanocyte-competent precursors, prevents patent ductus arteriosus. PLoS ONE.

[38] Liu JJ (2017). Regulation of dynein-dynactin-driven vesicular transport. Traffic.

[39] Palmisano I (2008). The ocular albinism type 1 protein, an intracellular G protein-coupled receptor, regulates melanosome transport in pigment cells. Hum. Mol. Genet..

[40] Lopes VS (2007). The ternary Rab27a-Myrip-Myosin VIIa complex regulates melanosome motility in the retinal pigment epithelium. Traffic.

[41] Coscoy S, Huguet E, Amblard F (2007). Statistical analysis of sets of random walks: how to resolve their generating mechanism. Bull. Math. Biol..

[42] Weiss M, Elsner M, Kartberg F, Nilsson T (2004). Anomalous subdiffusion is a measure for cytoplasmic crowding in living cells. Biophys. J..

[43] Saxton MJ (2007). A biological interpretation of transient anomalous subdiffusion. I. Qualitative model. Biophys. J..

[44] Brunstein M, Bruno L, Desposito M, Levi V (2009). Anomalous dynamics of melanosomes driven by myosin-V in *Xenopus laevis* melanophores. Biophys. J..

[45] Savin T, Doyle PS (2005). Static and dynamic errors in particle tracking microrheology. Biophys. J..

[46] Michalet X (2010). Mean square displacement analysis of single-particle trajectories with localization error: Brownian motion in an isotropic medium. Phys. Rev. E Stat. Nonlin. Soft Matter Phys..

[47] Schneider MA, Spoden GA, Florin L, Lambert C (2011). Identification of the dynein light chains required for human papillomavirus infection. Cell Microbiol..

[48] Apcarian A, Cunningham AL, Diefenbach RJ (2010). Identification of binding domains in the herpes simplex virus type 1 small capsid protein pUL35 (VP26). J. Gen. Virol..

[49] Chen XJ (2007). Proprioceptive sensory neuropathy in mice with a mutation in the cytoplasmic Dynein heavy chain 1 gene. J. Neurosci..

[50] Hafezparast M (2003). Mutations in dynein link motor neuron degeneration to defects in retrograde transport. Science.

[51] Courchesne SL, Pazyra-Murphy MF, Lee DJ, Segal RA (2011). Neuromuscular junction defects in mice with mutation of dynein heavy chain 1. PLoS ONE.

[52] Sabblah TT (2018). A novel mouse model carrying a human cytoplasmic dynein mutation shows motor behavior deficits consistent with Charcot-Marie-Tooth type 2O disease. Sci. Rep..

[53] Delmas V, Martinozzi S, Bourgeois Y, Holzenberger M, Larue L (2003). Cre-mediated recombination in the skin melanocyte lineage. Genesis.

[54] Chu Y (2012). Alterations in axonal transport motor proteins in sporadic and experimental Parkinson’s disease. Brain.

[55] Ye Q, Wen Y, Al-Kuwari N, Chen X (2020). Association between Parkinson’s disease and melanoma: putting the pieces together. Front. Aging Neurosci..

[56] Hu, H. H. et al. PARKIN Inactivation Links Parkinson’s disease to melanoma. *J. Natl Cancer Inst.* **108**, 10.1093/jnci/djv340 (2016). 10.1093/jnci/djv340 26683220

[57] Zhou L (2019). Effects of dynein light chain Tctex-type 3 on the biological behavior of ovarian cancer. Cancer Manag. Res..

[58] Rogers SL, Tint IS, Fanapour PC, Gelfand VI (1997). Regulated bidirectional motility of melanophore pigment granules along microtubules in vitro. Proc. Natl Acad. Sci. USA.

[59] Aspengren S, Wielbass L, Wallin M (2006). Effects of acrylamide, latrunculin, and nocodazole on intracellular transport and cytoskeletal organization in melanophores. Cell Motil. Cytoskelet..

[60] Frost R (2013). Acoustic detection of melanosome transport in *Xenopus laevis* melanophores. Anal. Biochem..

[61] Evans RD (2014). Myosin-Va and dynamic actin oppose microtubules to drive long-range organelle transport. Curr. Biol..

[62] Levi V, Serpinskaya AS, Gratton E, Gelfand V (2006). Organelle transport along microtubules in *Xenopus* melanophores: evidence for cooperation between multiple motors. Biophys. J..

[63] Mallik R, Gross SP (2004). Molecular motors: strategies to get along. Curr. Biol..

[64] Cordelieres FP (2013). Automated cell tracking and analysis in phase-contrast videos (iTrack4U): development of Java software based on combined mean-shift processes. PLoS ONE.

[65] Laurent-Gengoux, P. et al. Simulation of melanoblast displacements reveals new features of developmental migration. *Development* **145**, 10.1242/dev.160200 (2018). 10.1242/dev.160200 PMC6031402 29769218

[66] Pierzynska-Mach A, Janowski PA, Dobrucki JW (2014). Evaluation of acridine orange, LysoTracker Red, and quinacrine as fluorescent probes for long-term tracking of acidic vesicles. Cytom. A.

[67] Hurbain I, Romao M, Bergam P, Heiligenstein X, Raposo G (2017). Analyzing lysosome-related organelles by electron microscopy. Methods Mol. Biol..

