# Supplementary material for: A role for Dynlt3 in melanosome movement, distribution, acidity and transfer
Source: Commun Biol. 2021 Mar 26;4:423. doi: 10.1038/s42003-021-01917-5 (PMC7997999; doi:10.1038/s42003-021-01917-5)
Supplement: Supplementary file 1 — Supplementary Information [file 42003_2021_1917_MOESM1_ESM.pdf]

A role for Dynlt3 in melanosome movement, distribution, acidity and transfer

Zackie Aktary, Alejandro Conde-Perez, Florian Rambow, Mathilde Di Marco, François Amblard, Ilse Hurbain, Graça Raposo, Cédric Delevoye, Sylvie Coscoy & Lionel Larue

Supplementary Information

Supplementary Note 1

Analysis frame for Figure 6

The analysis was performed as previously described[1]. Briefly, we considered a general situation with diffusion, directional movement or tethering, with or without noise, represented by equation (1):

(1) $\mathbf{R}_{j+1} = \mathbf{R}_j + [\mathbf{D}].\boldsymbol{\delta}_j + \mathbf{V}\tau_0 - k_0(\mathbf{R}_j - \mathbf{R}_c)$

Elementary time steps of amplitude τ_0 led to time points t_j, such that $\tau_0 = t_{j+1} - t_j$, and $\mathbf{R}_j$ was the position at $t = t_j$. *dx* and *dy* steps in $\boldsymbol{\delta}_j$ were taken as independent gaussian variables. $[\mathbf{D}]$ was the diffusion matrix and $\mathbf{V} = (V_x, V_y)$ the velocity term. Noise due to experimental considerations like image analysis was represented by the term $A_{noise}\mathbf{g}_j$, where A_{noise} was the noise amplitude and $\mathbf{g}_j = (g_{x,j}, g_{y,j})$ representing normally distributed variables associated with the noise. The noise was added to each position $\mathbf{R}_j$. We also considered the following possible contributions: (i) a tethering term (k_0); (ii) a confined motion within a circle of radius

R, in which tethering was replaced by a reflective boundary condition; and (iii) intermittent diffusion (alternation of slow and fast diffusion). Details are given in [1].

In our general approach, the comparison between a set of experiments and a set of simulated trajectories was performed by fitting the distributions of eight descriptors related to the spatiotemporal and spatial characteristics of the trajectories. In this paper we used two main descriptors: the MSD exponent, and the second moments on the main long (μ_{2L}) and short (μ_{2S}) axes. The second moments refer only to 2D spatial characteristics and were obtained by projecting trajectory points on its own axis system, giving two unidimensional distributions which were statistically described by the computation of moments of order 2.

The Mean Square Displacement (MSD) was computed for $\tau = p\tau_0$ using $MSD(\tau) = \frac{\sum_{j \in J_p} (R_{p+j} - R_j)^2}{Card(J_p)}$. The set J_p of intervals (*j, j*+*p*), indexed by *j,* was taken as large as possible in the limit of independent intervals[2]. As a trade-off between good statistics and extended temporal dynamics, we limited the computation of the MSD for $\tau_0 \le \tau \le \tau_{\max}$, with $\tau_{\max} = T_{obs}/4$. $MSD(\tau)$ was fit with $b\tau^{\alpha}$, with a least square fit in the $(\log(t), \log(\langle r^2 \rangle))$ plane.

The analyses presented in this paper were not aimed to obtain detailed fits, but simply to evaluate the proportion of directional trajectories. These analyses were mainly based on the distributions: (i) of the MSD α exponent (Fig. 6) and (ii) on second-order moments (for homogeneity of subpopulations; heterogeneous subpopulations, i.e. with populations generated with different elementary processes, tend to widen these distributions). Details are given in [1].

Supplementary Note 2

Additional information linked to the analyses presented in Supplementary Figure 6

Panels (a-f) present the comparisons of WT *vs*. bcat* and WT-Ctrl (WT-GFP) *vs*. WT-bcat (WT-βcatGFP) for α distributions and (μ_{2L} and μ_{2S}).

Panels (g-i) present the MSD exponent distributions (left) and the normalized second moment distributions (right) for the [g] WT and bcat*, [h] WT-Ctrl and WT-bcat, and [i] WT-shCtrl and WT-shDynlt3 populations of trajectories. The Brownian mathematical reference is shown in black.

Panels (j-l) present the subdivisions of the melanosome movements into small (S) and large (L) amplitudes. For small-amplitude populations, α distributions were shifted to lower values compared to the Brownian reference (black line). Preliminary attempts revealed that adding elementary processes such as confinement and/or noise improved the fits. However, the aim of this work was not to establish the precise contribution of each of these components. The precise associated mechanism(s) may be more complex than a simple confinement mechanism in a circle and/or tethering to one unique binding site that we considered here, which may explain a wider α distribution. The mechanisms possibly involved were previously addressed in several publications on melanosome movement[3-5]. For large-amplitude populations, α distributions were shifted to the right, as expected for directional processes (examples of simulations with a single velocity are shown). However, α distributions were generally more broadly distributed, as would be expected with a single velocity, particularly for the "directional" conditions bcat* and WT-bcat. This might be explained by the fact that a wider distribution of velocities for the different trajectories is expected, rather than a unique velocity, possibly related to the different

numbers of motors involved[3]. After subdivision into small- and large-amplitude trajectories, a subdiffusive behaviour could be seen for small-amplitude trajectories. This could be indicative of a transient anomalous subdiffusion due to binding events[6]. In addition, the contribution of analysis noise may partly explain this effect at short time intervals [7].

For reference, classical MSD curves are represented in panel I for the different conditions. These curves correspond to computations of an average of trajectories. Large-amplitude trajectories showed mean behaviours suggesting a weak directional behaviour, more pronounced for bcat (bcat* and WT-bcat) and shDynlt3 trajectories than WT. This mean representation is completed by the distributions for individual trajectories presented in (i), showing the shift in α distributions corresponding to a directional movement generating process.

Supplementary References

1 Coscoy, S., Huguet, E. & Amblard, F. Statistical analysis of sets of random walks: how to resolve their generating mechanism. *Bulletin of mathematical biology* **69**, 2467-2492, doi:10.1007/s11538-007-9227-8 (2007).

2 Flyvbjerg, H. & Petersen, H. G. Error-estimates on averages of correlated data. *J Chem Phys* **91**, 461-466 (1989).

3 Levi, V., Serpinskaya, A. S., Gratton, E. & Gelfand, V. Organelle transport along microtubules in Xenopus melanophores: evidence for cooperation between multiple motors. *Biophys J* **90**, 318-327, doi:10.1529/biophysj.105.067843 (2006).

4 Brunstein, M., Bruno, L., Desposito, M. & Levi, V. Anomalous dynamics of melanosomes driven by myosin-V in Xenopus laevis melanophores. *Biophys J* **97**, 1548-1557, doi:10.1016/j.bpj.2009.06.048 (2009).

5 Bruno, L., Salierno, M., Wetzler, D. E., Desposito, M. A. & Levi, V. Mechanical properties of organelles driven by microtubule-dependent molecular motors in living cells. *PLoS One* **6**, e18332, doi:10.1371/journal.pone.0018332 (2011).

6 Saxton, M. J. A biological interpretation of transient anomalous subdiffusion. I. Qualitative model. *Biophys J* **92**, 1178-1191, doi:10.1529/biophysj.106.092619 (2007).

7 Martin, D. S., Forstner, M. B. & Kas, J. A. Apparent subdiffusion inherent to single particle tracking. *Biophys J* **83**, 2109-2117, doi:10.1016/S0006-3495(02)73971-4 (2002).

Supplementary Figure Legends

Supplementary Figure 1. Altered localization of Tyrp1 melanosomes in melanocytes overexpressing β-catenin or with decreased levels of Dynlt3. Immunofluorescence analysis of 5 different cells in each of the different melanocyte cell lines with either increased levels of β-catenin or decreased levels of Dynlt3. Cells were processed for immunofluorescence 48 hours after transfection and stained with anti-Tyrp1 (red) antibodies. Nuclei were stained with DAPI (blue). Bar, 15 μm.

Supplementary Figure 2. Melanosomes are distributed at the periphery of melanocytes expressing exogenous activated β-catenin.

a) Brightfield, inverse brightfield and GFP images of WT (9v) melanocytes transiently expressing GFP or GFP-tagged β-catenin. Bar, 20 μm.

b) Quantification of the total number of pigmented melanosomes in WT melanocytes transiently expressing GFP or GFP-tagged β-catenin. Results represent the mean ± the standard deviation (SD) of pooled data from three independent experiments with a total of twelve cells for each transfection. ns signifies no statistical significance ($p = 0.561$) as determined by the two-sided Mann-Whitney test.

c) Quantification of the number of perinuclear pigmented melanosomes in WT melanocytes transiently expressing GFP or GFP-tagged β-catenin. Results represent the mean ± SD of pooled data from three independent experiments with a total of twelve cells for each transfection. Statistical significance was determined by the two-sided Mann-Whitney test and **** signifies $p < 0.0001$.

d) Immunofluorescence analysis of WT melanocytes transiently expressing GFP or GFP-tagged β-catenin. Cells were processed for immunofluorescence 48 hours

after transfection and stained with anti-Tyrp1 (red) antibodies. Nuclei were stained with DAPI (blue). In Supplementary Fig. 1, more examples are shown. Bar, 20 μm.

Supplementary Figure 3. bcat* melanosomes are larger than WT melanosomes.

a,b) Transmission electron microscopy of WT (a) and bcat* (b) melanocytes. Scale bar: 2 μm.

c) Melanosome length was determined from four WT and five bcat* melanocytes with a total of at least 200 melanosomes per cell line. Statistical significance was measured using a two-sided Mann-Whitney test. **** $p < 0.0001$.

d) Melanosome area was determined from four WT and five bcat* melanocytes with a total of at least 200 melanosomes per cell line, using the formula for the area of an ellipse ($A = Lw\pi$). Statistical significance was measured using a two-sided Mann-Whitney test. **** $p < 0.0001$.

Supplementary Figure 4. Scans of the blots presented in Aktary et al. Scans of western blot panels shown in Figures 2c, 2f, 2j, 2n, 3b and 5b are presented, with the bands of interest highlighted.

Supplementary Figure 5. Dynlt3, and not Dynlt1, is expressed in WT and downregulated in bcat* melanocytes.

Ct values for RT-qPCR analysis of Dynlt3 (a) and Dynlt1 (b) mRNA expression in two cell lines derived from WT mice (9v and 13d) and two from bcat* mice (10d and 14d). Each experiment was repeated three times from three consecutive passages. Error bars represent the SD. Statistical analysis was performed using an unpaired, two-

sided T-test. ns means non-significant (p-values were equal or superior to 0.4590), ** p = 0.0047.

Supplementary Figure 6. Melanosome movement is increased in cells producing active β-catenin or with diminished level of Dynlt3.

Melanosome movement (corresponding mainly to stage IV melanosomes) was assessed by brightfield video microscopy over a period of 5 minutes and melanosome trajectories were followed using *ImageJ* software. For each cell line, 75 melanosomes were followed from a minimum of nine independent cells. WT cell lines were transfected with control GFP or β-catenin-GFP expression vectors, respectively. Analyses were performed on both transfected (i.e. GFP-positive) and non-transfected (i.e. GFP-negative) cells. In addition, WT cells were transfected with either control or Dynlt3 shRNA vectors and analyses were performed on the resulting red cells. Each dot represents one melanosome. The average distance (a) and the average velocity (b) for each melanosome are shown, with the error bars representing the SD. The percentages of centripetal (c) and centrifugal (d) movements of the melanosomes are shown. Statistical significance was measured using two-sided Mann-Whitney tests (a,b) and X^2 tests (c,d). ns = non-significant ($p = 0.4992$), * $p = 0.0293$, ** $p = 0.0037$ (WT vs. bcat*) and 0.0027 (WT-GFP vs. WT-βcat-GFP) , **** $p < 0.0001$.

Supplementary Figure 7. Mathematical modeling of melanosome trajectories.

a,d) α distributions for WT and bcat* (a), and WT-Ctrl (WT-GFP) and WT-bcat (WT-βcatGFP) (d) populations of trajectories. (D), directional, and (ND) non-directional.

b,c,e,f) μ_{2S}, μ_{2L} plots showing the subpopulations of WT (b), bcat* (c), WT-Ctrl (e), and WT-bcat (f) trajectories. Each point corresponds to one trajectory. Lines correspond to frontiers between non-directional (ND) and directional (D) trajectories.

g,h,i) α MSD and L normalized second moment distributions for WT and bcat* (g), WT-Ctrl and WT-bcat (h), and WT-shCtrl and WT-shDynlt3 (i) populations of trajectories. Brownian motion, a mathematical reference, is shown as a black line (n_{walks}=10,000, n_{steps}=601).

j) Criteria used to establish the $\mu_{2S}.\mu_{2L}$ frontiers for non-directional (ND) and directional (D) movement for α MSD and second moment distributions (L and S). $\mu_{2S}.\mu_{2L}$ frontiers define small-amplitude (black) and large-amplitude (dashed grey) populations of trajectories. The Brownian reference is indicated in thick black, with constraints being (1) μ_2 distributions after subdivision should not be more extended than the Brownian reference (if not possible for both, at least for small-amplitude populations); (2) large-amplitude populations should be directional (right shifted compared to the reference). These criteria were met for a range of values $\mu_{2S}.\mu_{2L}$ 1.4×10^{-2} to 2.2×10^{-2} μm^4 (with same lower and upper frontiers for all conditions). α and μ_{2L} distributions after subdivision with the middle frontier ($\mu_{2S}.\mu_{2L}$ 1.8×10^{-2} μm^4) are shown here, and were very close to distributions with lower and upper frontiers (not shown), which defined an error bar for our subdivision.

k) Comparison of α distributions after subdivision with simple generating processes. Top, small-amplitude populations. α distributions are shown for control small-amplitude populations on the left (WT [A], blue, WT-Ctrl [C], green, WT-shCtrl [F], grey), and corresponding “mutant” conditions on the right (bcat* [H]; blue, WT-bcat

[D]; green, and WT-shDynlt3 [G]; grey). Theoretical distributions are shown for the Brownian mathematical reference (black), a confined process (red, with $R_{confinement}/(4D.\tau_0)^{1/2}$=12.5), or the addition of noise (magenta, with $A_{noise}/(4D.\tau_0)^{1/2}$=0.5). Bottom, large-amplitude populations, same colour code as in the top panel for controls (left) and corresponding “mutant” conditions (right). Theoretical distributions are shown for the Brownian mathematical reference (black) and a directional process (red, with $V\tau_0/(4D.\tau_0)^{1/2}$=0.05).

l) Mean Square Displacements (mean of trajectories). Top, trajectories of total populations. Middle (small-amplitude) and bottom (large-amplitude), trajectories of the different subpopulations after the subdivision described in (j). Black, slope α=1 corresponding to a diffusive process. (α>1 for directional process, α<1 for subdiffusion). WT [A]; blue, WT-Ctrl [C]; green, WT-shCtrl [F]; grey, bcat* [H]; red, WT-βcat [D]; magenta, and WT-shDynlt3 [G]; orange.

For all panels, WT-Ctrl refers to WT-GFP cells and WT-bcat refers to WT-βcat-Δex3-GFP cells.

Supplementary Figure 8. Assessment of melanosome acidity by Acridine Orange staining. Typical photomicrographs showing the number of acidic melanosomes in WT, bcat*, WT-siCtrl and WT-siDynlt3 melanocytes that were treated with 20 μg/mL Acridine Orange 20 minutes at 37°C. Each micrograph represents approximately three fields used for quantifications. The cells were then incubated in warm PBS and imaged using a confocal microscope. Melanosome acidity was assessed by counting the number of pigmented melanosomes that were positive for acridine orange staining. Bar, 5 μm.

Supplementary Figure 9. Confocal immunofluorescence microscopy and FACS analysis of melanosome transfer.

a) Confocal immunofluorescence analysis of melanosome transfer between WT or bcat* melanocytes and Balb/c MK keratinocytes. Melanocytes and keratinocytes were co-cultured for 10 days, fixed and processed for immunofluorescence analyses using Plakoglobin (shown in magenta as a false colour) and Hmb45 (shown in green) antibodies. The yellow arrows indicate Pmel positive staining localized outside of melanocytes. Bar, 30 μm.

b) Immunofluorescence analysis of melanosome transfer between melanocytes (WT + sh Ctrl or WT + shDynlt3) and Balb/c MK keratinocytes. Melanocytes and keratinocytes were co-cultured for 10 days, fixed and processed for immunofluorescence analyses using Plakoglobin (shown in magenta as a false colour) and Hmb45 (shown in green) antibodies. The yellow arrows indicate Pmel positive staining localized outside of melanocytes.

c) Gating strategy for FACS experiments. Melanocyte and keratinocyte cells were stained with either control IgG, plakoglobin or Pmel antibodies. The live cell population was chosen for gating. IgG control antibodies were used to establish a baseline of background signal. Following this, the established gate was applied to samples stained with the respective antibodies of interest. The gates were kept constant for all cell lines (melanocyte and keratinocyte) and for the co-culture experiments.

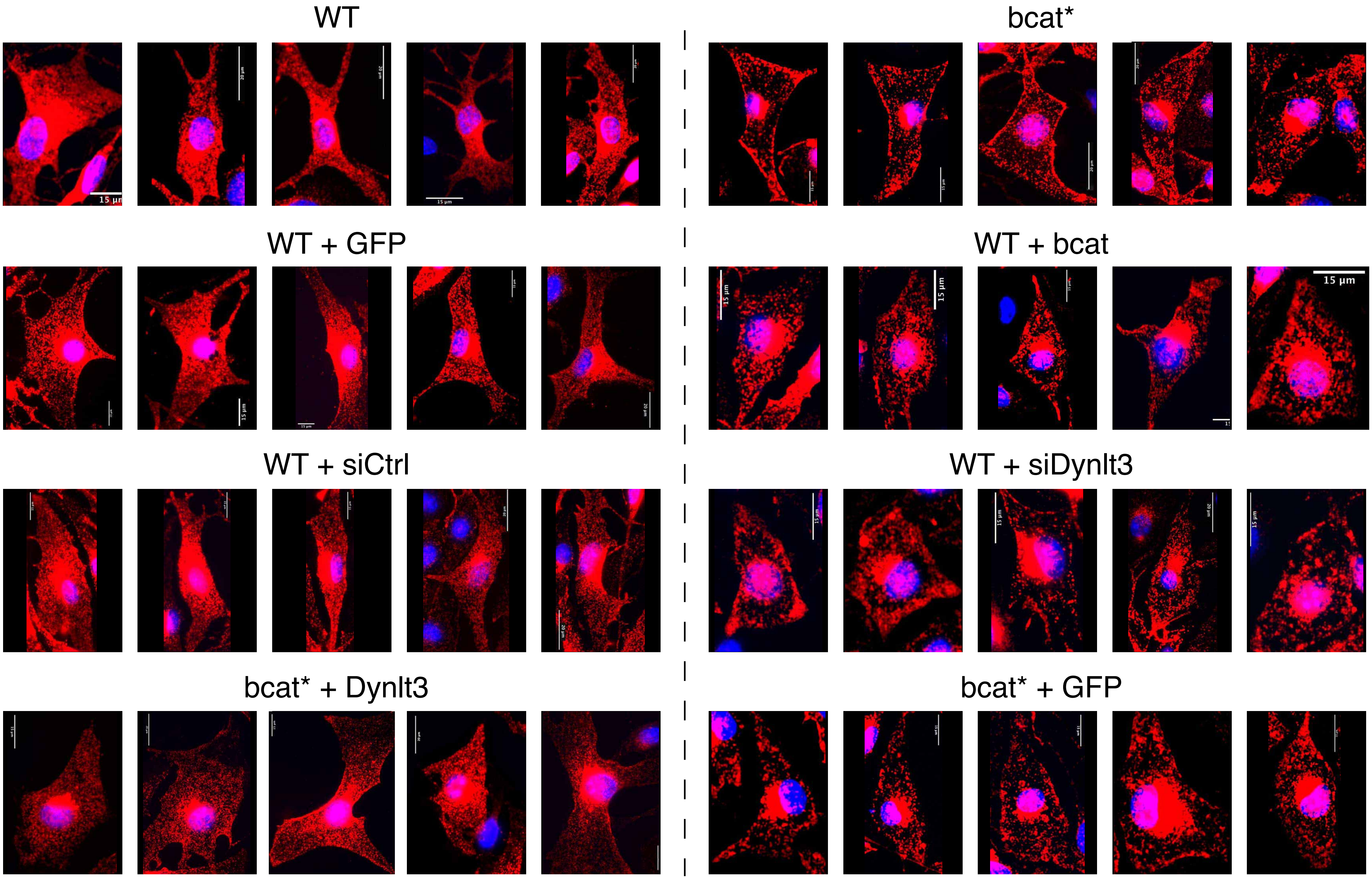

Supplementary Figure 1

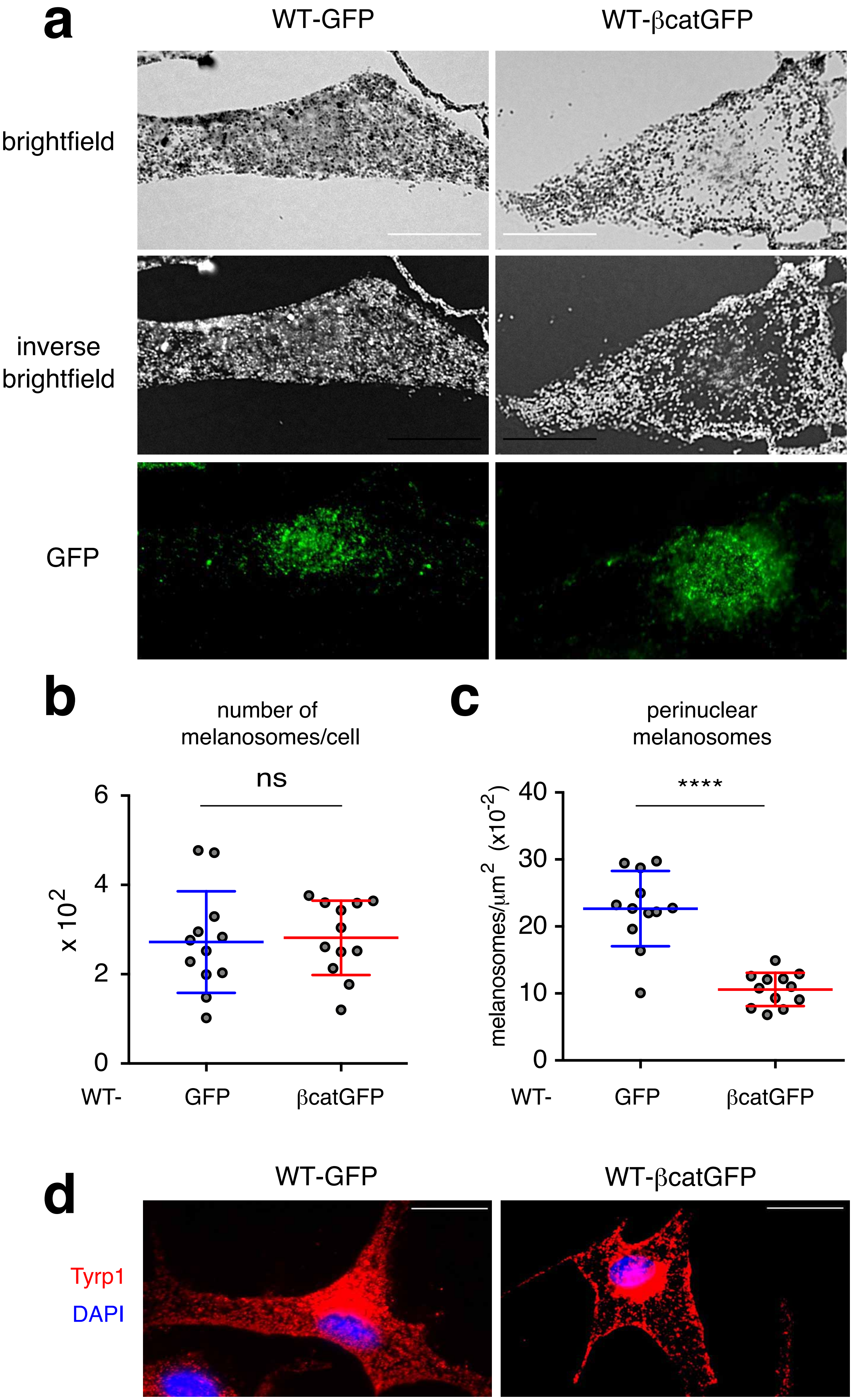

Supplementary Figure 2

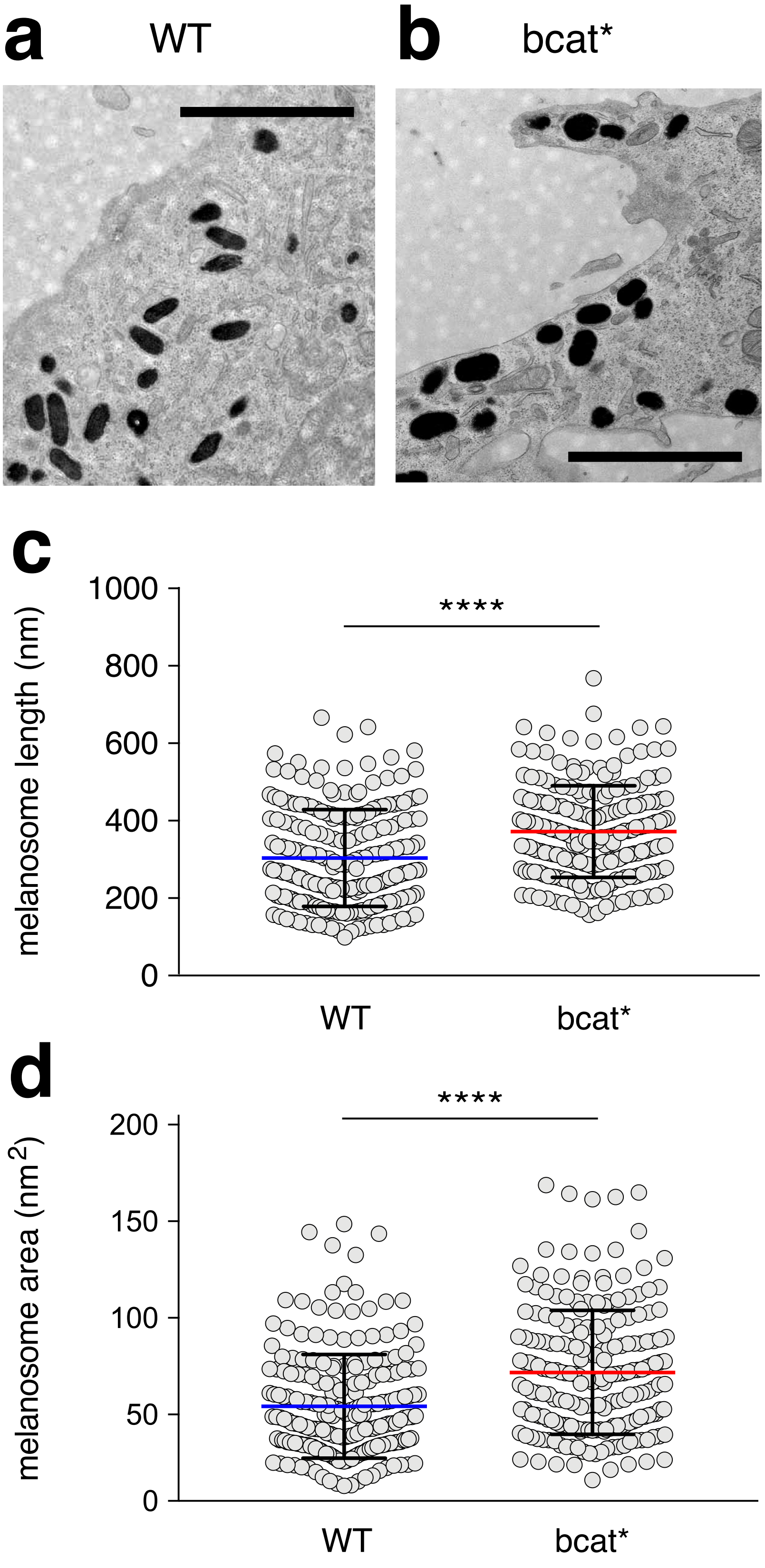

Supplementary Figure 3

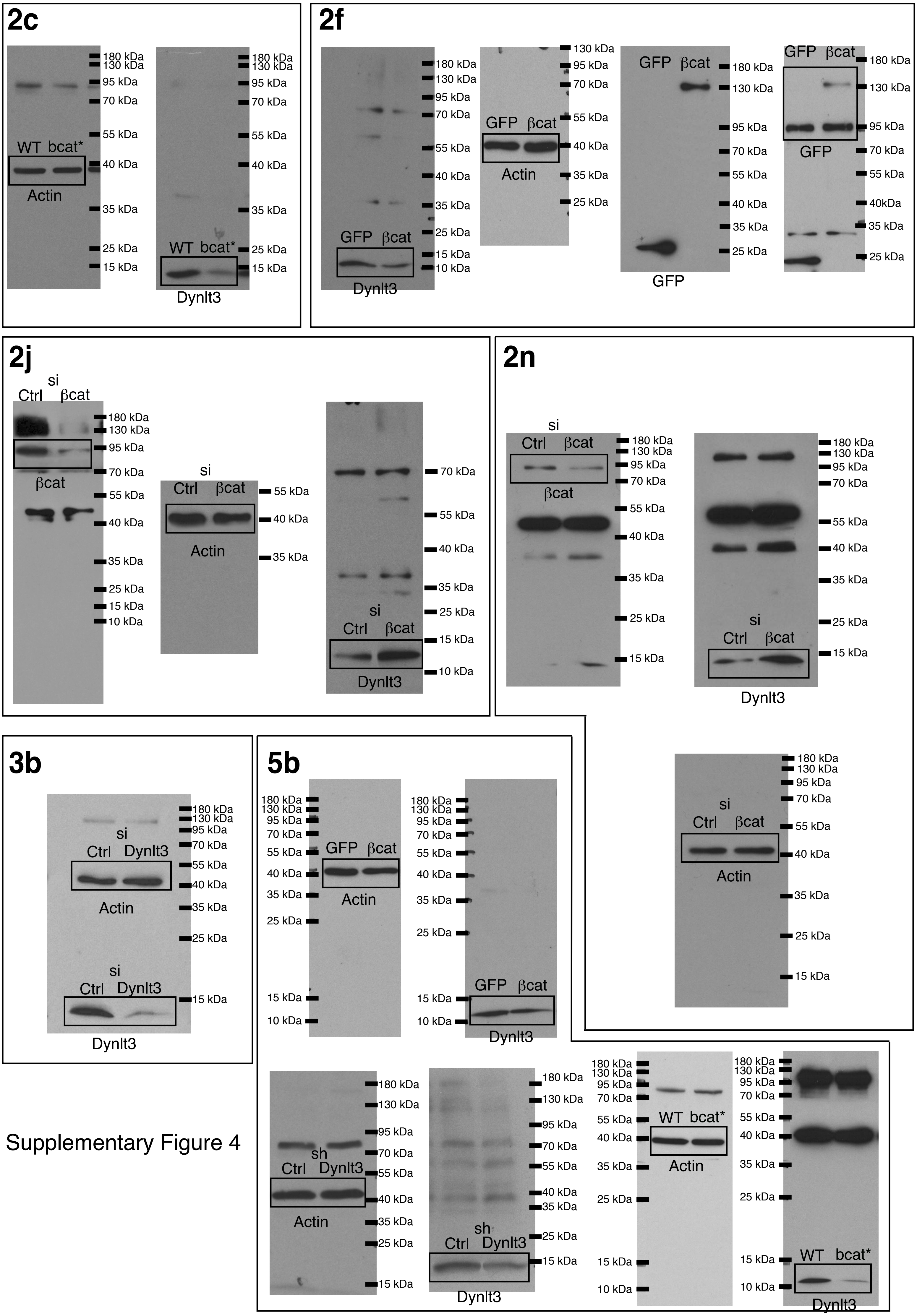

Supplementary Figure 4

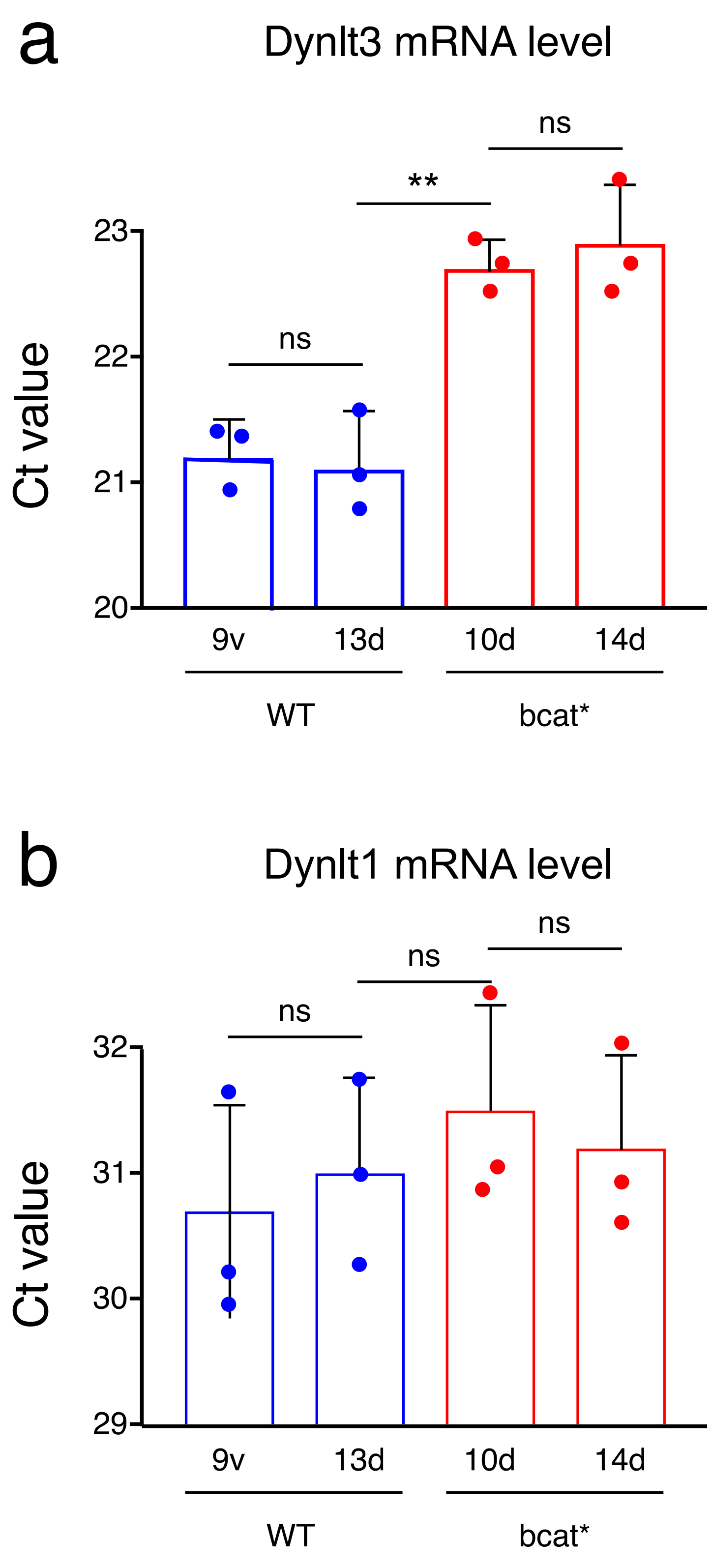

Supplementary Figure 5

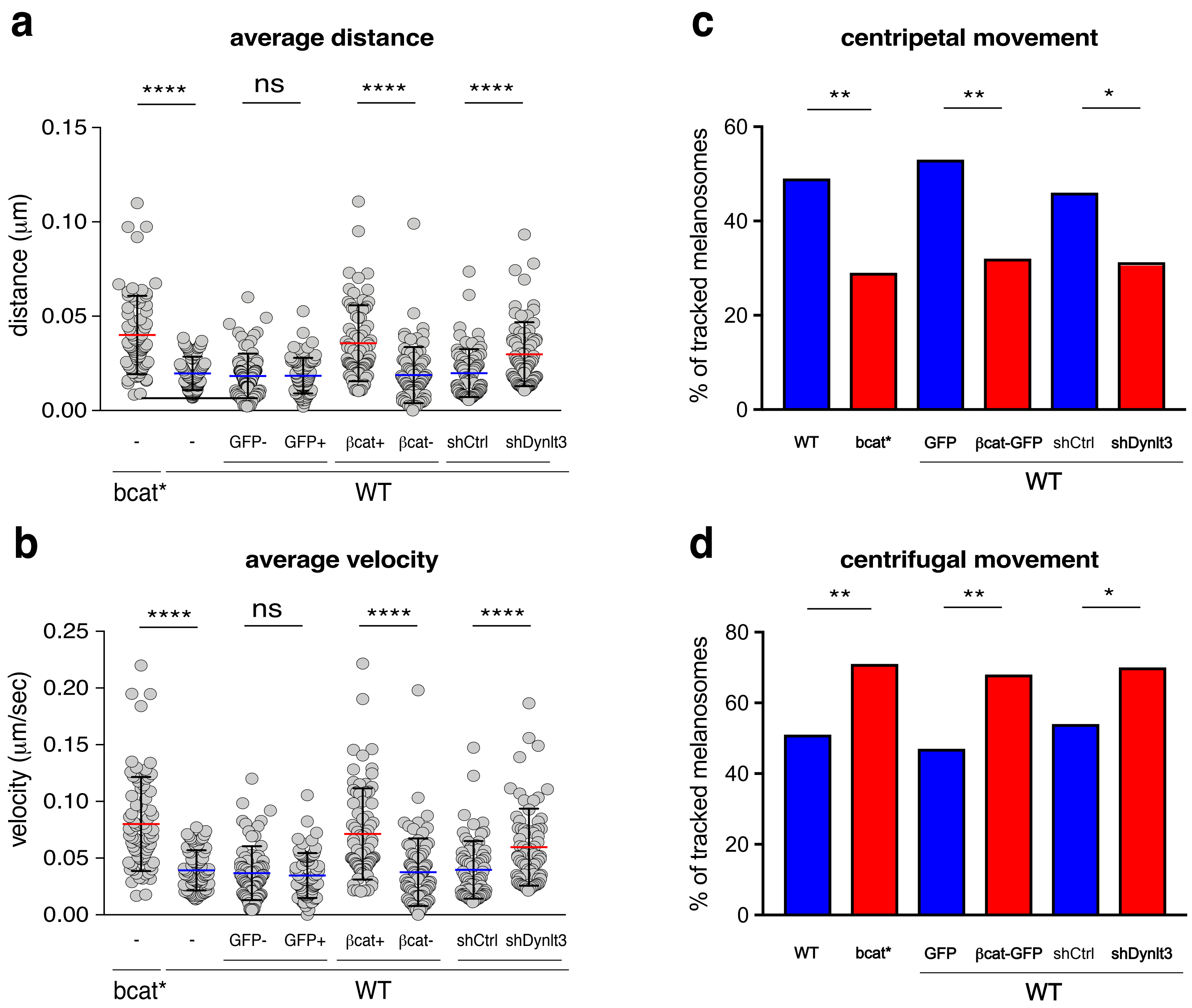

Supplementary Figure 6

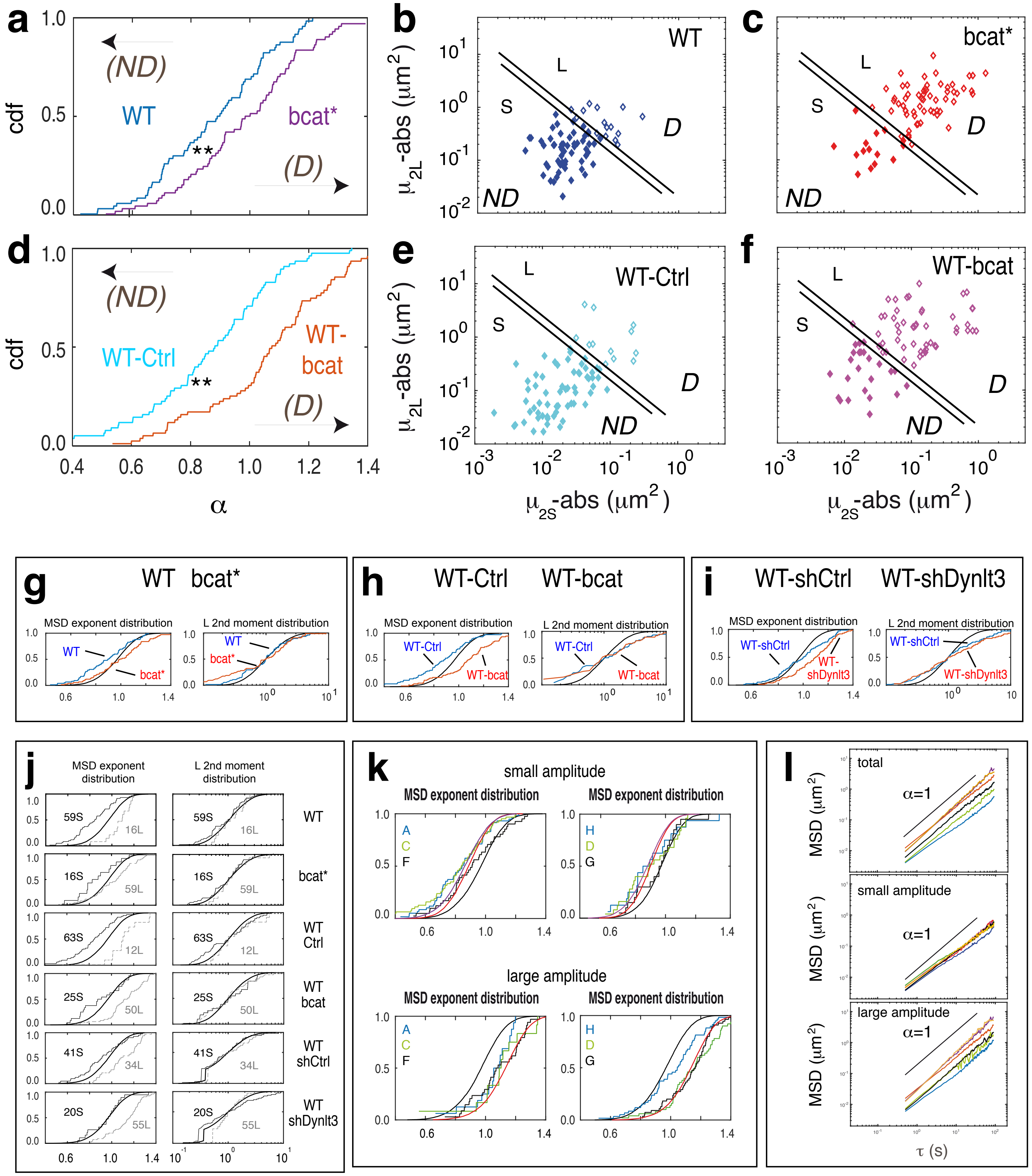

Supplementary Figure 7

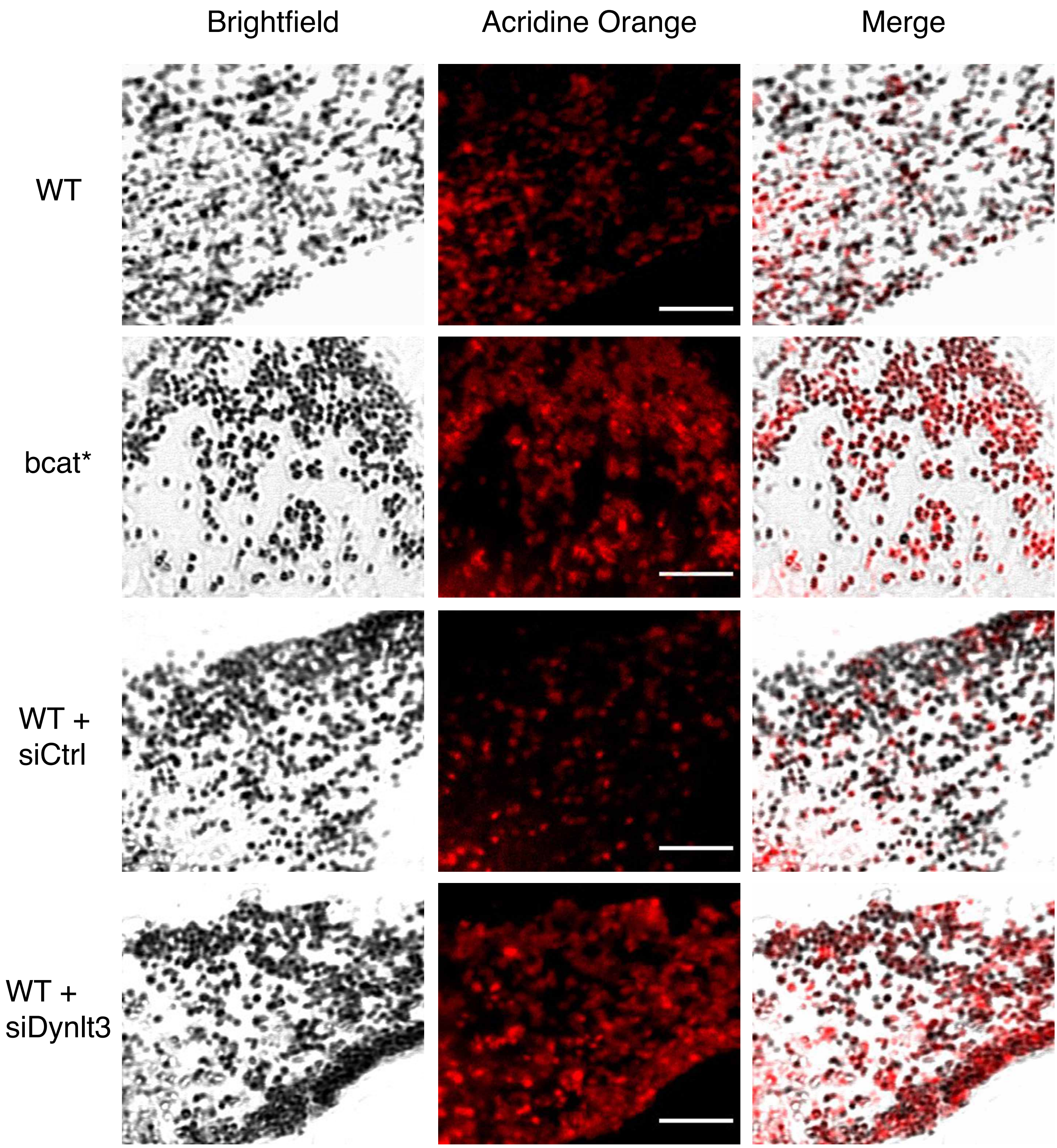

Supplementary Figure 8

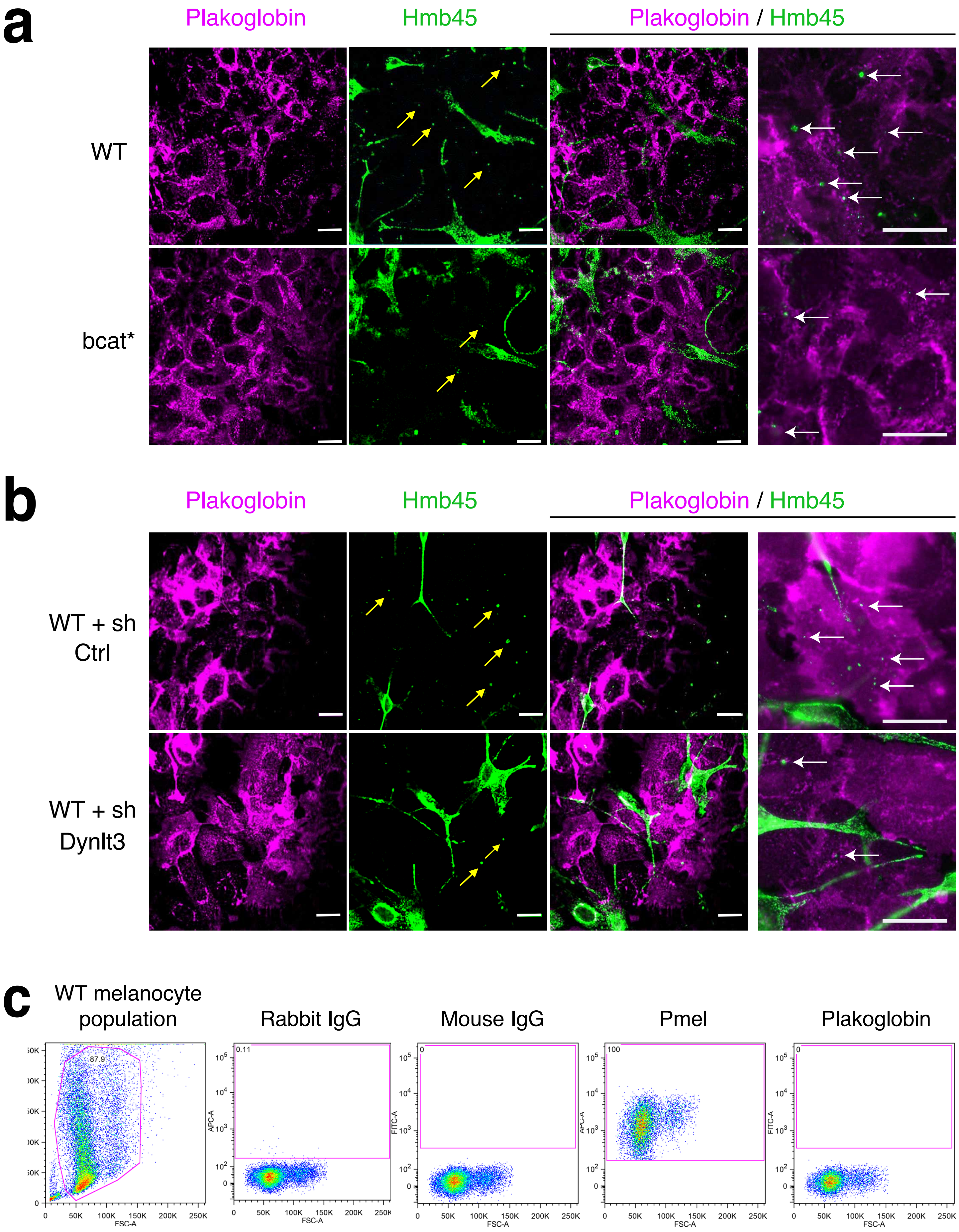

Supplementary Figure 9
