# Supplementary material for: A role for Dynlt3 in melanosome movement, distribution, acidity and transfer
Source: Commun Biol. 2021 Mar 26;4:423. doi: 10.1038/s42003-021-01917-5 (PMC7997999; doi:10.1038/s42003-021-01917-5)
Supplement: Supplementary file 2 — Description of Supplementary Files [file 42003_2021_1917_MOESM2_ESM.pdf]

Description of Additional Supplementary Files

File name: Supplementary Movie 1

Description: Video of melanosome movement in WT cells. WT melanocytes were recorded for a period of five minutes, with one image being taken every 0.5 seconds, for a total of 601 images. Bar, 20 μm.

File name: Supplementary Video 2

Description: Video of melanosome movement in bcat* cells. bcat* melanocytes were recorded for a period of five minutes, with one image being taken every 0.5 seconds, for a total of 601 images. Bar, 20 μm.

File name: Supplementary Data 1

Description: Mouse models associated with mutations in 67 genes involved in trafficking of melanosomes and other lysosome related organelles (LRO). Notes: 1- no conditional mutant mice available; 2- absence of mouse model; 3- conditional mutant mice available. Inactivation in the melanocyte lineage was not performed yet.

File name: Supplementary Data 2

Description: Numerical source data for the graphs and charts presented in the main figures of Aktary et al.
