# Supplementary material for: A role for Dynlt3 in melanosome movement, distribution, acidity and transfer
Source: Commun Biol. 2021 Mar 26;4:423. doi: 10.1038/s42003-021-01917-5 (PMC7997999; doi:10.1038/s42003-021-01917-5)
Supplement: Supplementary file 7 — Reporting Summary [file 42003_2021_1917_MOESM7_ESM.pdf]

nature research

Corresponding author(s): Larue
Last updated by author(s): Feb 22, 2021

Reporting Summary

Nature Research wishes to improve the reproducibility of the work that we publish. This form provides structure for consistency and transparency in reporting. For further information on Nature Research policies, see our Editorial Policies and the Editorial Policy Checklist.

Statistics

For all statistical analyses, confirm that the following items are present in the figure legend, table legend, main text, or Methods section.

n/a	Confirmed	
☐	☒	The exact sample size (*n*) for each experimental group/condition, given as a discrete number and unit of measurement
☐	☒	A statement on whether measurements were taken from distinct samples or whether the same sample was measured repeatedly
☐	☒	The statistical test(s) used AND whether they are one- or two-sided<br>*Only common tests should be described solely by name; describe more complex techniques in the Methods section.*
☒	☐	A description of all covariates tested
☒	☐	A description of any assumptions or corrections, such as tests of normality and adjustment for multiple comparisons
☐	☒	A full description of the statistical parameters including central tendency (e.g. means) or other basic estimates (e.g. regression coefficient) AND variation (e.g. standard deviation) or associated estimates of uncertainty (e.g. confidence intervals)
☒	☐	For null hypothesis testing, the test statistic (e.g. *F*, *t*, *r*) with confidence intervals, effect sizes, degrees of freedom and *P* value noted<br>*Give P values as exact values whenever suitable.*
☒	☐	For Bayesian analysis, information on the choice of priors and Markov chain Monte Carlo settings
☒	☐	For hierarchical and complex designs, identification of the appropriate level for tests and full reporting of outcomes
☒	☐	Estimates of effect sizes (e.g. Cohen's *d*, Pearson's *r*), indicating how they were calculated

Our web collection on statistics for biologists contains articles on many of the points above.

Software and code

Policy information about availability of computer code

Data collection	No code was used for data collection in this study.
Data analysis	No code was used for data analysis in this study.

For manuscripts utilizing custom algorithms or software that are central to the research but not yet described in published literature, software must be made available to editors and reviewers. We strongly encourage code deposition in a community repository (e.g. GitHub). See the Nature Research guidelines for submitting code & software for further information.

Data

Policy information about availability of data

All manuscripts must include a data availability statement. This statement should provide the following information, where applicable:

- Accession codes, unique identifiers, or web links for publicly available datasets
- A list of figures that have associated raw data
- A description of any restrictions on data availability

The Affymetrix transcriptomic data generated during this study have been deposited in the NCBI GEO and are available under the accession number GSE167268. All numerical source data for the main figures are included in this published article’s Supplementary Data 2 file.

Field-specific reporting

Life sciences study design

All studies must disclose on these points even when the disclosure is negative.

Sample size	For western blot and RT-PCR analyses, we used at least three independent biological experiments. For tracking experiments of melanosomes, we used at least 50 melanosomes from at least 5 cells for each point.
Data exclusions	No data were excluded, except for one outlier that was excluded in figure 5C. It is mentioned in the legend.
Replication	Each experiment was performed at least three independent times to verify the results.
Randomization	Tracked melanosomes were randomly taken as long as they were pigmented.
Blinding	The measurements of Figs 1b, 1c, 3c, 3d, 5, and Figs S2b, S2c, S3 and S5 were performed blindly by two different individuals.

Reporting for specific materials, systems and methods

We require information from authors about some types of materials, experimental systems and methods used in many studies. Here, indicate whether each material, system or method listed is relevant to your study. If you are not sure if a list item applies to your research, read the appropriate section before selecting a response.

Materials & experimental systems

n/a	Involved in the study	
☐	☒	Antibodies
☐	☒	Eukaryotic cell lines
☒	☐	Palaeontology and archaeology
☐	☒	Animals and other organisms
☒	☐	Human research participants
☒	☐	Clinical data
☒	☐	Dual use research of concern

Methods

n/a	Involved in the study	
☒	☐	ChIP-seq
☐	☒	Flow cytometry
☒	☐	MRI-based neuroimaging

Antibodies

Antibodies used	They are included in the manuscript
Validation	We used the antibodies according to the manufacturer information, previous articles, and/or si RNA experiment validations.

Eukaryotic cell lines

Policy information about cell lines

Cell line source(s)	Mouse melanocyte cell lines were generated in the laboratory and the mouse keratinocyte cell line was obtained from ATCC.
Authentication	Mouse cell lines were published in Delmas et al. 2007, Gallagher et al. 2013, Petit et al., 2019.. Keratinocytes are validated by ATCC.
Mycoplasma contamination	All cell lines were regularly tested for Mycoplasma and were all negative.
Commonly misidentified lines (See ICLAC register)	No commonly misidentified cell lines were used in this study.

Animals and other organisms

Policy information about studies involving animals; ARRIVE guidelines recommended for reporting animal research

Laboratory animals	Male and female transgenic mice have the same phenotype, The presented mice are two-months old.
Wild animals	No wild animals were used in this study.
Field-collected samples	No field-collected samples were used in this study.
Ethics oversight	Animal care and use for this study were performed in accordance with the recommendations of the European Community (2010/63/UE) for the care and use of laboratory animals. Experimental procedures were specifically approved by the ethics committee of the Institut Curie CEEA-IC #118 (CEEA-IC 2016-001) in compliance with the international guidelines.

Note that full information on the approval of the study protocol must also be provided in the manuscript.

Flow Cytometry

Plots

Confirm that:

- [x] The axis labels state the marker and fluorochrome used (e.g. CD4-FITC).
- [x] The axis scales are clearly visible. Include numbers along axes only for bottom left plot of group (a 'group' is an analysis of identical markers).
- [x] All plots are contour plots with outliers or pseudocolor plots.
- [x] A numerical value for number of cells or percentage (with statistics) is provided.

Methodology

Sample preparation	Flow cytometry was done on mouse melanocyte-keratinocyte co-cultures. Cells were fixed in PFA, permabilized with saponin, and stained for specific antibodies of interest, as described in the materials and methods section.
Instrument	BD FACSCanto II
Software	FlowJo
Cell population abundance	The entire cell population was used for analyses.
Gating strategy	Gating was done by first using an appropriate IgG control to identify background signal. This gate was then used to determine cell positivity for each antibody in each cell line. These gates were then used for the co-culture experiments.

- [x] Tick this box to confirm that a figure exemplifying the gating strategy is provided in the Supplementary Information.
